# OsteoNet-X: A deep residual learning framework for automated osteoporosis detection from knee X-ray images

**DOI:** 10.1371/journal.pone.0361033

**Published:** 2026-10-27

**Authors:** Sudhir Kumar Sangula, S. P. Siddique Ibrahim

**Affiliations:** 1 School of Computer Science and Engineering, VIT-AP University, Amaravati, Andhra Pradesh, India; 2 School of Computer Science and Engineering, VIT-AP University, Amaravati, Andhra Pradesh, India; University of Vermont, UNITED STATES OF AMERICA

## Abstract

Osteoporosis is a progressive skeletal disorder characterized by reduced bone mineral density and increased susceptibility to fractures, making early diagnosis essential for effective clinical intervention and long-term patient management. Recent advances in artificial intelligence have demonstrated significant potential for automated bone disease assessment using medical imaging modalities. In this study, a unified deep learning framework, termed OsteoNet-X, is proposed for automated osteoporosis detection and multi-class bone health classification using knee X-ray images. Unlike conventional image classification approaches, OsteoNet-X integrates YOLO-based Region of Interest (ROI) localization, DenseNet-based feature extraction, Feature-Aware Learning (FAL) optimization, and Grad-CAM-based Explainable Artificial Intelligence (XAI) to enhance both classification performance and model interpretability. The ROI extraction stage automatically identifies anatomically relevant knee joint regions, reducing background interference and enabling more discriminative feature learning. Subsequently, DenseNet facilitates efficient feature propagation and hierarchical representation learning for distinguishing normal, osteopenia, and osteoporosis conditions. The incorporation of XAI further provides visual explanations of model decisions by highlighting clinically relevant regions within the knee joint. Experimental evaluation demonstrates that OsteoNet-X achieves a classification accuracy of 97.08%, a macro F1-score of 96.61%, and a weighted F1-score of 97.11%.

## 1. Introduction

Osteoporosis is a gradual systemic skeletal illness that raises the risk of fracture and bone fragility. It is characterised by microarchitectural degeneration of bone tissue and loss of bone mass, it has an estimated 200 million people afflicted worldwide, osteoporosis is one of the most common non-communicable illnesses impacting ageing populations, according to the World Health Organization (WHO) [1]. Osteoporotic fractures, particularly in those involving the hip and knee-adjacent regions, are associated with substantial morbidity, mortality, and long-term disability, placing a significant socioeconomic burden on healthcare systems [2]. Epidemiological researchers found that in every three women and in every five men over the age of 50 one would have an osteoporotic fracture throughout their lifetime [3]. Early diagnosis of osteoporosis is critical and also as timely intervention can significantly reduce fracture risk and slow disease progression. Dual-energy X-ray absorptiometry (DXA) is the standard for bone mineral density (BMD) assessment, with diagnostic thresholds defined by T-score and Z-score measurements [4]. But the DXA scanners are very expensive, it requires specialized infrastructure, and are not widely accessible in low- and middle-income regions. Moreover, DXA-based screening is often underutilized, leading to delayed diagnosis until fractures occur [5]. There are continuous interest in opportunistic screening approaches that leverage routinely acquired medical images, such as plain radiographs, for osteoporosis risk assessment.

Knee X-ray imaging is one of the most common radiological procedures performed globally every year. Recent clinical evidence indicates that structural variations in the trabecular bone patterns surrounding the knee joint, specifically in the proximal tibia and distal femur, are rich in information about bone mineral density and osteoporosis severity [6]. Nonetheless, these patterns are usually very subtle, localized, and hard to extract using conventional image processing and manual radiological analysis. There is, therefore, a great need to automate and develop data-driven approaches to extract informative features about osteoporosis from knee radiographs.

Recently, deep learning, especially convolutional neural networks (CNNs), has achieved outstanding performance in medical image analysis tasks, such as disease diagnosis, staging, and prediction [7]. ResNet [8], DenseNet [9], and EfficientNet [10], which are CNN-based models, have been extensively used because of their capability to learn hierarchical features from raw image inputs. Various research works have been conducted on the application of deep learning for osteoporosis diagnosis from spine, hip, and hand radiographs, and promising outcomes have been obtained [11,12]. However, the existing methods have one or more of the following drawbacks:

(i) reliance on small, homogeneous datasets,
(ii) limited focus on knee-specific osteoporosis patterns,
(iii) poor generalization under severe class imbalance, and
(iv) lack of explainability, which is a critical requirement for clinical adoption.

Another emerging challenge in osteoporosis classification is the multi-stage nature of disease progression, typically categorized into Normal, Osteopenia, and Osteoporosis. While the binary classification (osteoporotic vs. non-osteoporotic) is useful for screening, the accurate multi-class severity grading is essential for treatment planning and risk stratification [13]. Overlapping visual traits across neighboring classes and significant class imbalance in real-world datasets make multi-class classification extremely difficult.

Despite achieving a high degree of numerical accuracy, these deep learning models continue to operate as “black boxes,” giving a very little information about how decisions are made. Their practical deployment is severely hampered by their interpretability, as doctors and radiologists require visual confirmation of the model’s predictions to boost trust and guarantee patient safety [14]. Explainable artificial intelligence (XAI) techniques such as Gradient-weighted Class Activation Mapping (Grad-CAM) have been demonstrated to be effective in revealing diagnostically significant regions of medical images [15]. To close the current gap between model performance and usability, XAI must be included into osteoporosis classification algorithms.

In this work, we utilize a clinically annotated knee osteoporosis dataset available in Mendeley “Knee X-ray Osteoporosis Database” with 240 knee X-ray images annotated at three levels of severity (Normal, Osteopenia, and Osteoporosis), along with 239 corresponding clinical records containing demographic, anthropometric, and bone density-related variables [16]. The dataset is prone to large real-world challenges such as class imbalance, small sample size, and varying distributions of features, making it an ideal test bed for assessing robust and real-world models, the sample of the samples are shown in Fig 1. Thorough exploratory data analysis (EDA) was performed to know more into the distributions of features, demographic patterns, and relationships between features.

**Fig 1:**
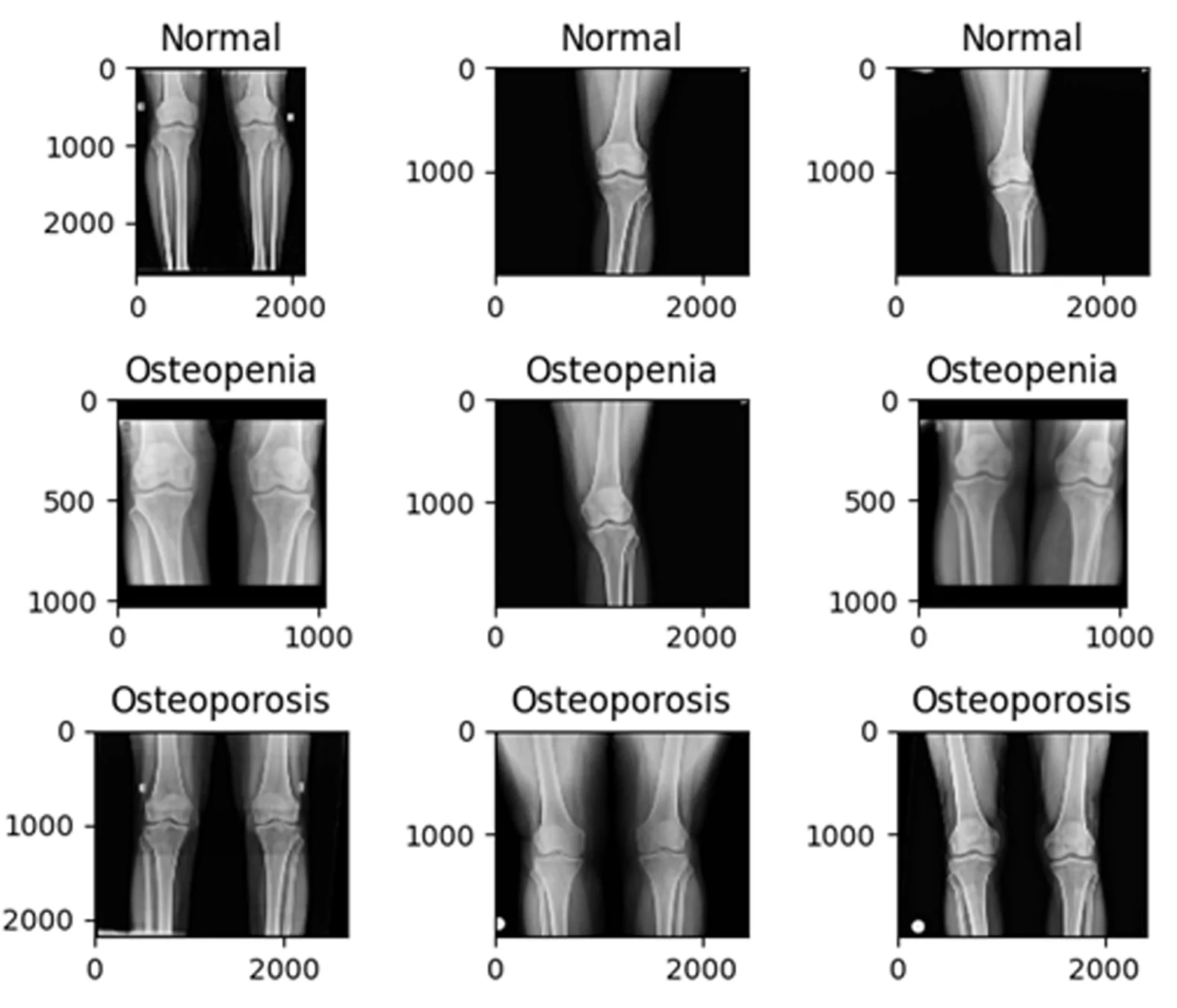
Images of knee X-ray samples from each diagnostic category in the dataset.

Fig 1 shows some example knee X-ray images belonging to each of the diagnostic classes (normal, osteopenia, and osteoporosis), which convey the visual differences and variations that exist among the classes. Although knee radiographs are becoming more prevalent in clinical settings, currently, there is no standardized, automated, and interpretable approach for accurate osteoporosis severity level using knee X-ray images classification. Existing deep learning models either target binary classification without considering severity levels, are not robust in class imbalance situations, or are not interpretable for clinical acceptance. Furthermore, simple fusion of clinical and imaging modalities tends to result in performance deterioration because of overfitting and dominance problems, especially when working with small datasets. Therefore, there is an urgent need for developing a stable, image-focused, and interpretable deep learning model that can effectively classify osteoporosis severity levels.

This study presents OsteoNet-X, a unified deep learning framework for automated osteoporosis severity classification from knee X-ray images. Unlike conventional end-to-end image classification models, OsteoNet-X integrates multiple complementary components designed specifically for medical image analysis. The framework combines YOLO-based Region of Interest (ROI) localization for automatic extraction of anatomically relevant knee joint regions, DenseNet-based feature learning for robust hierarchical representation extraction, Feature-Aware Learning (FAL) optimization to enhance discriminative feature utilization, and Grad-CAM-based Explainable Artificial Intelligence (XAI) for clinical interpretability. By focusing the learning process on diagnostically significant bone regions and employing dense feature propagation mechanisms, the proposed framework aims to improve classification robustness while reducing the influence of irrelevant image structures and background noise.

It is important to note that the ResNet-based model presented in this study serves exclusively as a baseline architecture for comparative evaluation. The final OsteoNet-X framework extends beyond conventional residual learning by incorporating YOLO-based ROI extraction, DenseNet-based feature learning, Feature-Aware Learning optimization, and Grad-CAM explainability. This distinction is maintained throughout the experimental and ablation analyses to clearly demonstrate the contribution of each component to the overall performance of the proposed framework.

The primary objective of this study is to develop and evaluate an accurate, robust, and interpretable deep learning framework for multi-class osteoporosis severity assessment using knee radiographic images. Specifically, the proposed OsteoNet-X framework seeks to address the limitations of existing osteoporosis classification systems by integrating ROI-guided localization, dense feature extraction, feature-aware optimization, and explainable decision-making within a unified architecture. Furthermore, the study investigates the contribution of each architectural component through comprehensive ablation analysis and comparative experimentation against conventional image classification approaches. Through extensive evaluation, OsteoNet-X is demonstrated as a clinically relevant computer-aided diagnostic framework capable of supporting automated osteoporosis screening, severity grading, and explainable decision support in medical imaging environments.

The remainder of this paper is organized as follows. Section 2 reviews related work on osteoporosis detection and knee Radiograph analysis using a deep learning approach Section 3 describes the dataset, preprocessing pipeline, and exploratory data analysis. Section 4 presents the proposed OsteoNet-X architecture and overall methodology. Section 5 outlines the experimental setup and evaluation metrics used for performance assessment. Section 6 presents experimental results and comparative analysis. Finally, Section 7 concludes the paper and highlights the potential direction for future research.

## 2. Related work

The systemic skeletal illness known as osteoporosis is characterised by reduced bone mass and defects in microarchitecture that raise the risk of fractures. Dual-energy X-ray absorptiometry is costly and not always accessible, which has been the basis for the traditional diagnosis of osteoporosis [17,18]. The use of deep learning and other artificial intelligence algorithms to identify osteoporosis on traditional radiography has drawn interest [19].

### 2.1. Deep learning for osteoporosis from X-Ray imaging

CNNs and transfer learning have been studied for the purpose of identifying osteoporosis in X-ray images. Using VGG-16, ResNet-50, and DenseNet-121, Qureshi et al. [20] developed a transfer learning model that outperformed earlier models with shallower architectures in classifying knee X-rays into three groups. Similar to this, separate research employing many pre-trained models showed that sophisticated CNNs can classify knee and hip X-ray images with excellent accuracy, underscoring the promise of deep feature extraction for bone pathology investigation Gunde [21]. These methods, however, generally ignore the larger patient context in favour of concentrating just on imaging findings.

### 2.2. Multimodal models with clinical data integration

The recent studies emphasize the importance of integrating imaging information with clinical and demographic information to obtain better performance. A study done by Tang et al. [22] integrated clinical information and features from chest X-ray images using deep learning to obtain better performance in the detection of osteoporosis, obtaining fewer false negatives than that from image features only. Another systematic review highlights the need to integrate clinical risk factors (age, BMI, fracture history) with radiographic features to enhance generalizability and clinical utility of AI models Zhang et al. [23]. These approaches are consistent with clinical practice, where imaging information and risk factors are considered together.

### 2.3. Explainability in osteoporosis AI models

Techniques for Explainable AI have emerged as a response to the “black-box” problem of deep learning models in the healthcare domain. In addition to accuracy, XAI techniques have enabled the interpretation of decisions made by models. For example, comparative analyses evaluate the use of various frameworks for explainability (LIME, permutation feature importance, SHAP) to interrogate the predictions of osteoporosis risk, showing that transparent models can confirm the importance of variables such as age and hormonal changes. More recent work in prototype-based multimodal architectures provides explainability by design, offering interpretable visual and prototype explanations for bone health classification Pellicer et al.[24],.

### 2.4. Advanced and hybrid methodologies

Beyond straightforward CNN classification, hybrid frameworks and feature fusion strategies have been explored to solve the problems such as limited annotated data and model interpretability. In addition to traditional deep learning, researchers have proposed multi-modal fusion combined with variable clustering and PCA dimensionality reduction to enhance interpretability and performance by jointly considering imaging and clinical patterns Zubair et al.[25],. Ensemble approaches and multi-scale fusion models have also been applied to improve robustness against data imbalance and feature variability Jain et al.[26],.

### 2.5. Data privacy and collaborative learning

Whereas many such studies have relied on centralized datasets, recent work in federated learning provides a means to collectively train models on multiple institutions without sharing underlying patient data, thus maintaining privacy while still benefiting from the larger data distribution Pati et al.[27],. Though federated learning has been extensively explored for general medical imaging tasks, its exploration for osteoporosis classification is still in its infancy and serves as one of the primary motivations for this work.

Table 1 summarizes some recent methodologies for knee osteoarthritis (KOA) detection and severity grading using radiographic, clinical, and multimodal data. The reviewed studies span a range of techniques, including traditional convolutional neural networks, transfer learning, fine-grained deep learning, multimodal fusion, and systematic reviews. Most research use publicly accessible datasets like MOST and the Osteoarthritis Initiative (OAI), although some employ proprietary clinical or multiomic databases for validation.

**Table 1:** Summary of some existing studies on knee osteoarthritis detection and severity assessment.

Authors	Methodology	Dataset Used	Performance	Limitations
Cueva et al. [28] (2022)	Semi-automatic CADx system using Deep Siamese CNN with fine-tuned ResNet-34 for bilateral knee analysis	Public OA dataset (training); private clinical dataset (validation)	Average multi-class accuracy of 61%; better performance for KL-0, KL-3, KL-4; validated against radiologists	Poor discrimination for intermediate grades (KL-1, KL-2); moderate overall accuracy
Mohammed et al. [29] (2023)	Transfer learning with six pretrained CNNs (VGG16/19, ResNet101, MobileNetV2, InceptionResNetV2, DenseNet121)	Osteoarthritis Initiative (OAI) X-ray dataset	Best results with ResNet101: 69% (5-class), 83% (binary), 89% (3-class)	Reduced performance for full 5-class grading; dependence on dataset configuration
Toyooka et al. [30] (2023)	Retrospective radiographic analysis using CPAK classification (aHKA + JLO)	500 knees from Japanese TKA patients	Type I phenotype most prevalent (53.8%); consistent sex-matched distribution	Not a DL-based study; population-specific, limited generalizability
Goswami [31] (2023)	CNN-based classification with image sharpening preprocessing	OAI baseline knee X-ray images	Mean accuracy of 91.03% for 5-grade KL classification; 19% improvement over non-enhanced images	Focuses mainly on preprocessing; early-grade sensitivity not deeply analyzed
Rani et al. [32] (2024)	Custom 12-layer CNN for binary and multi-class KOA classification	OAI knee X-ray dataset	92.3% accuracy (binary); 78.4% accuracy (KL multi-class)	Lower performance for severity grading compared to binary task; limited external validation
Lee et al. [33] (2024)	Fine-grained DL using pixel-level Plug-In Modules (PIMs); ensemble of four models	OAI (training); MOST dataset (17,040 images testing)	High accuracy for KL-4 (96%); strong generalization	Low accuracy for KL-1 (43%); early-stage OA remains challenging
Liew et al. [34] (2023)	Scoping review of early-stage KOA definitions across literature	211 studies from PubMed, EMBASE, Cochrane, Web of Science	Identified high heterogeneity; KL grading most common criterion	No unified definition of early-stage KOA; frequent inclusion of KL ≥ 2
Diab et al. [35] (2025)	Collective transfer learning + feature selection using binary Greylag Goose Optimizer; DL + ML classifiers	KOA imaging dataset (not explicitly named)	Accuracy: 98.87%; Sensitivity: 98.01%; Specificity: 99.01%; statistically validated	High model complexity; optimization-driven methods may affect reproducibility
Rockel et al. [36] (2025)	Multimodal deep learning (VAE + K-means) integrating multiomic biofluid data	414 KOA patients (plasma, synovial fluid, urine)	Identified 3 KOA endotypes; improved WOMAC outcome prediction post-TKA	Requires costly multiomic data; limited clinical scalability
Zhao et al. [37] (2025)	Systematic review and meta-analysis of DL-based KOA X-ray classification	19 studies; 62,158 knee X-ray images	High sensitivity for KL-4 (90.32%); moderate for KL-0,2,3	Sensitivity for early-stage KOA (KL-1) remains inadequate

The reported performance indicates that deep learning models achieve strong results for binary classification and advanced disease stages (KL-3 and KL-4). However, some persistent issues remain in the early KOA diagnosis and multi-class severity level classification. Some more recent studies have attempted to focus on multimodal and optimization-based approaches for enhanced diagnostic performance, but often at the cost of increased model complexity. Notably, the table above emphasizes the progress and the remaining challenges in the existing frameworks for KOA assessment.

### 2.6. Common limitations identified in existing studies

Despite the progress made in the use of deep learning and multimodal approaches for the detection and severity estimation of knee osteoarthritis, some common drawbacks have been observed in the existing literature. One of the most prominent drawbacks that have been widely reported in the literature is the lack of discrimination between the early and intermediate stages of knee osteoarthritis, specifically Kellgren-Lawrence grades KL-1 and KL-2. Although the existing approaches have shown high accuracy in the estimation of the severity of advanced stages of knee osteoarthritis, such as KL-3 and KL-4, their sensitivity in capturing the minute structural changes associated with early-stage knee osteoarthritis is still not satisfactory.

Another common drawback that has been observed in the existing literature is the class imbalance problem and dataset bias, particularly in large public databases such as the Osteoarthritis Initiative (OAI). As a result, models often demonstrate reduced generalizability across the full spectrum of disease severity, particularly for underrepresented classes. Many other studies depend on imaging data alone, with limited incorporation of clinical and demographic variables such as age, body mass index, fracture history, and hormonal factors, despite their well-established clinical relevance in osteoarthritis risk assessment.

The lack of use of explainability remains a critical barrier to clinical adoption, several number of DL models are black boxes, which offer excellent predictive results but lack interpretability. The lack of interpretability of these models hinders their acceptance by clinicians and prevents them from being used in clinical settings and most of the current models are based on centralized training paradigms, which involve the aggregation of patient data at a central location. This has raised concerns about data security and privacy.

The limitations concerning external validation, model complexity, and reproducibility are common. The ability to perform well on internal validation sets is shown in several studies, but generalization to other sets or populations is not shown. Moreover, computational cost and reproducibility problems are often caused by frameworks that rely on optimization, ensemble models, and highly complex architectures, which are less practical for real-world applications.

The recurrent methodological and clinical limitations noted in recent research on knee osteoarthritis and bone health assessment using radiographic and DL-based techniques are compiled in Table 2. Instead of listing the problems on a per-study basis, the table groups multiple authors under shared limitation categories, thereby highlighting systematic research gaps that persist across different datasets, model architectures, and evaluation settings. Reduced sensitivity to early-stage disease, poor performance in multi-class severity grading, reliance on class imbalance and dataset configuration, high computational complexity, and restricted external generalisability are some of the main drawbacks. The table also highlights more general issues like the dependence on expensive or non-routine clinical data and the absence of standardised definitions for early-stage KOA.

**Table 2:** Commonly observed limitations across existing KOA and bone health studies.

Authors	Models/ Approaches Involved	Common Limitations
Cueva et al.; Lee et al.; Zhao et al.; Goswami	Siamese CNN (ResNet-34); Fine-grained DL with PIMs; CNN with image enhancement; DL-based KL grading frameworks	Poor performance in early-stage disease (KL-1/ intermediate grades)
Mohammed et al.; Rani et al.; Cueva et al.	Transfer learning CNNs (ResNet, VGG, DenseNet); Custom CNN architectures; Bilateral DL models	Reduced accuracy in multi-class severity grading compared to binary tasks
Mohammed et al.; Zhao et al.; Lee et al.	Pretrained CNNs; Ensemble DL models; Multi-dataset evaluation frameworks	Strong dependence on dataset configuration and class imbalance
Lee et al.; Diab et al.; Rockel et al.	Ensemble DL with PIMs; Optimization-driven DL–ML hybrids; Multimodal VAE-based models	High model complexity and computational overhead
Rani et al.; Toyooka et al.; Goswami	Custom CNNs; Radiographic phenotype analysis; Single-cohort DL studies	Limited external validation or population generalizability
Liew et al.; Zhao et al.	Literature reviews; Meta-analytical DL assessments	Lack of standardized definition for early-stage KOA
Rockel et al.; Diab et al.	Multiomic DL pipelines; Optimization-heavy hybrid systems	Reliance on non-routine or costly clinical data
Goswami; Diab et al.	Image enhancement–driven CNNs; Metaheuristic-optimized feature pipelines	Over-reliance on preprocessing or handcrafted optimization strategies

### 2.7. How the proposed OsteoNet-X model addresses existing limitations (novelty and contributions)

The proposed framework is designed to overcome the limitations observed in existing deep learning-based osteoporosis and knee radiograph analysis systems. Particularly those related to instability under small datasets, poor multi-class discrimination, and lack of clinically meaningful interpretability. OsteoNet-X used a simplified yet highly discriminative end-to-end convolutional learning paradigm, designed for radiographic bone texture analysis different to a number of previous methods that rely on intricate region proposal mechanisms, multimodal fusion strategies, or aggressive cross-validation schemes.

#### 2.7.1. Robust feature learning without explicit ROI dependency.

The reliance on explicit anatomical region-of-interest extraction techniques like object detection or segmentation, which frequently introduce error propagation and sensitivity to annotation quality, is a big problem seen of earlier research. In solution to this, OsteoNet-X uses deep hierarchical residual feature learning, which enables the network to directly and implicitly learn discriminative bone patterns from complete knee X-ray images. Without the need for manually created or detector-driven ROIs, the model captures both local cortical irregularities and global trabecular distribution using stacked residual blocks and progressive receptive field expansion. This design maintains high sensitivity to structural degradation associated with osteoporosis while eliminating ROI localisation failure modes.

#### 2.7.2. Stability under limited and imbalanced clinical data.

Conventional cross-validation strategies, although theoretically appealing, were empirically shown to produce unstable and misleading performance under the dataset conditions used. OsteoNet-X, on the other hand, emphasises training stability and generalisation by:

- Medical-style transfer learning with pretrained ResNet backbones,
- Controlled layer freezing and gradual fine-tuning,
- Class-weighted optimization to counter severe class imbalance.

By avoiding repeated fold-based resampling and focusing on a clinically realistic train–test paradigm, the proposed model achieves substantially higher and more consistent multi-class classification performance, particularly for underrepresented osteoporosis cases. This directly addresses the reproducibility concerns reported in earlier osteoporosis imaging studies.

#### 2.7.3. Effective deep residual representation for multi-class severity assessment.

Most of the previous research shows only moderate success when switching from binary (normal vs. abnormal) categorisation to clinically meaningful multi-class grading (normal, osteopenia, osteoporosis). By taking use of deep residual feature reuse, OsteoNet-X specifically addresses this problem and allows the network to learn fine-grained inter-class boundaries. The residual formulation lessens gradient deterioration and makes it easier to learn subtle radiographic variations between adjacent disease stages, which is important for early osteoporosis detection and progression tracking.

#### 2.7.4. Integrated and clinically aligned explainability.

One major obstacle to the application of DL models in healthcare settings is the absence of explainable decision-making. Class-wise localisation of the discriminative bone region is made possible by OsteoNet-X’s smooth integration of the Grad-CAM-based visual explainability approach into the evaluation procedure. Grad-CAM in OsteoNet-X offers pathophysiologically correct information on activation areas, such as subchondral bone areas and trabecular density areas, in contrast to other shallow visualisation techniques. This increases the clinician’s trust in the model and facilitates validation.

#### 2.7.5. Lightweight, reproducible, and clinically deployable design.

Unlike ensemble-rich or optimization-centric architectures, OsteoNet-X focuses on architectural simplicity with high discriminative ability. The architecture uses a single deep CNN architecture, standardized preprocessing, and deterministic training settings for:

- Reproducibility across runs,
- Reduced computational overhead,
- Practical feasibility for deployment in clinical imaging workflows.

Comparative analysis against another backbones demonstrates that OsteoNet-X achieves superior performance through architecture-aware fine-tuning rather than architectural complexity.

**Contributions**: The novelty of this study’s OsteoNet-X lies in its evidence-driven architectural refinement, where only empirically validated components are retained. The key contributions include:

- A robust end-to-end residual learning framework for multi-class osteoporosis detection from knee X-rays.
- Elimination of fragile ROI and multimodal dependencies while maintaining high discriminative accuracy.
- A stability-focused training strategy tailored for small, imbalanced medical datasets.
- Integrated Grad-CAM explainability aligned with radiological reasoning.
- A reproducible, lightweight, and clinically deployable deep learning pipeline.

## 3. Methodology

### 3.1. Dataset description

Knee X-ray images and associated clinical information for osteoporosis diagnosis make up the multimodal dataset used in this study, data is organized in a way that allows for analysis of bone health conditions, namely Normal, Osteopenia, and Osteoporosis. The image dataset consists of 240 knee X-ray images, divided into three class-specific directories. Each image is used for supervised learning and represents a different patient and the corresponding clinical dataset starts with 243 patient records that include medical risk factors, anthropometric data, bone density indicators, and demographic information. After preprocessing and inter-modal alignment, only 239 patient records were selected, each specifically paired with a corresponding X-ray image through a normalized patient identifier. This guarantees a one-to-one correspondence between the image and clinical data, precluding data leakage or inappropriate supervision.

### 3.2. Clinical data preprocessing

The clinical data preprocessing was done in a structured multi-stage pipeline that is built to make data consistency, semantic correctness, and alignment with imaging data.

#### 3.2.1. Column name standardization.

The raw clinical data has a varying conventions in the column naming, which included spaces and special characters, and to make it compatible with the subsequent machine learning process, the column names were normalized.

This process involved:

- Trimming leading and trailing whitespace
- Replacing spaces with underscores
- Removing special characters such as colons and parentheses

This step ensures robust feature referencing and eliminates syntactic ambiguity during model development. The data-standardization workflow is illustrated in Fig 2.

**Fig 2:**
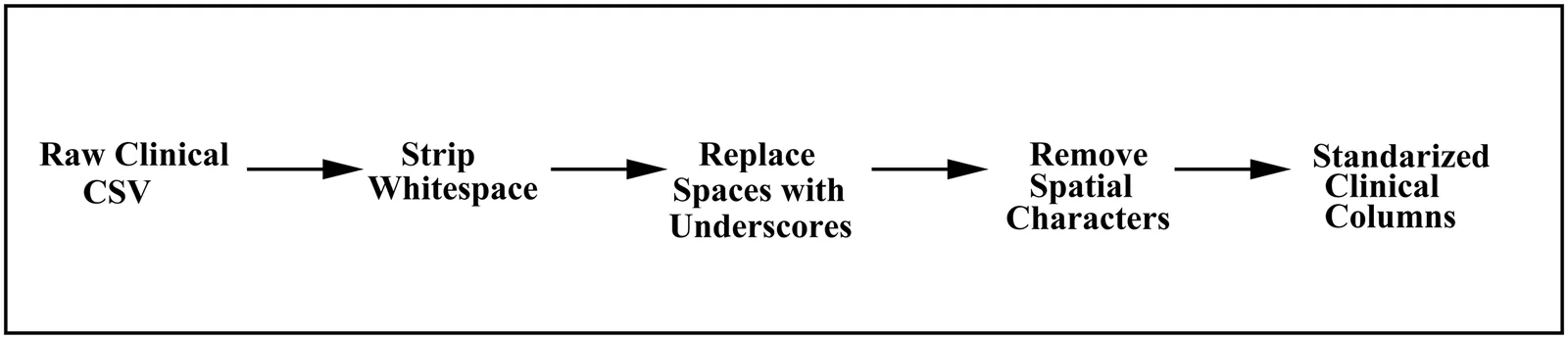
Data standardization process.

#### 3.2.2. Label encoding.

Each category of diagnosis was given a discrete integer label based on a clinically meaningful ordinal scale, and a numerical class label was generated from the diagnostic field to enable supervised learning.

- Normal → 0
- Osteopenia → 1
- Osteoporosis → 2

This encoded label serves as the ground truth for multi-class classification tasks. The label-encoding procedure is summarized in Fig 3.

**Fig 3:**
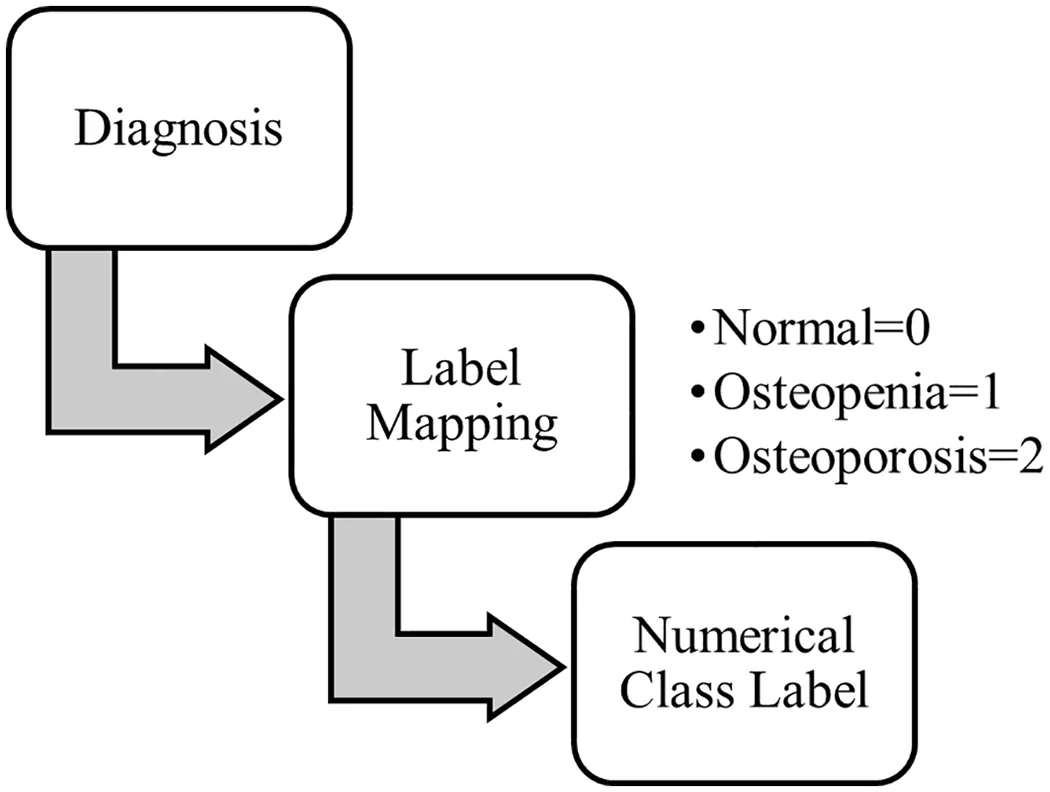
Label encoding process.

#### 3.2.3. Image-clinical record matching.

In order to attain multimodal consistency, image identifiers were derived from the filenames in every class directory, and patient identifiers from the clinical dataset were matched with image filenames. Particularly, images without patient records and patient records without images were found and examined. Due to variations in patient identifier formats, this step displayed some inconsistencies. The image–clinical record matching procedure is shown in Fig 4.

**Fig 4:**
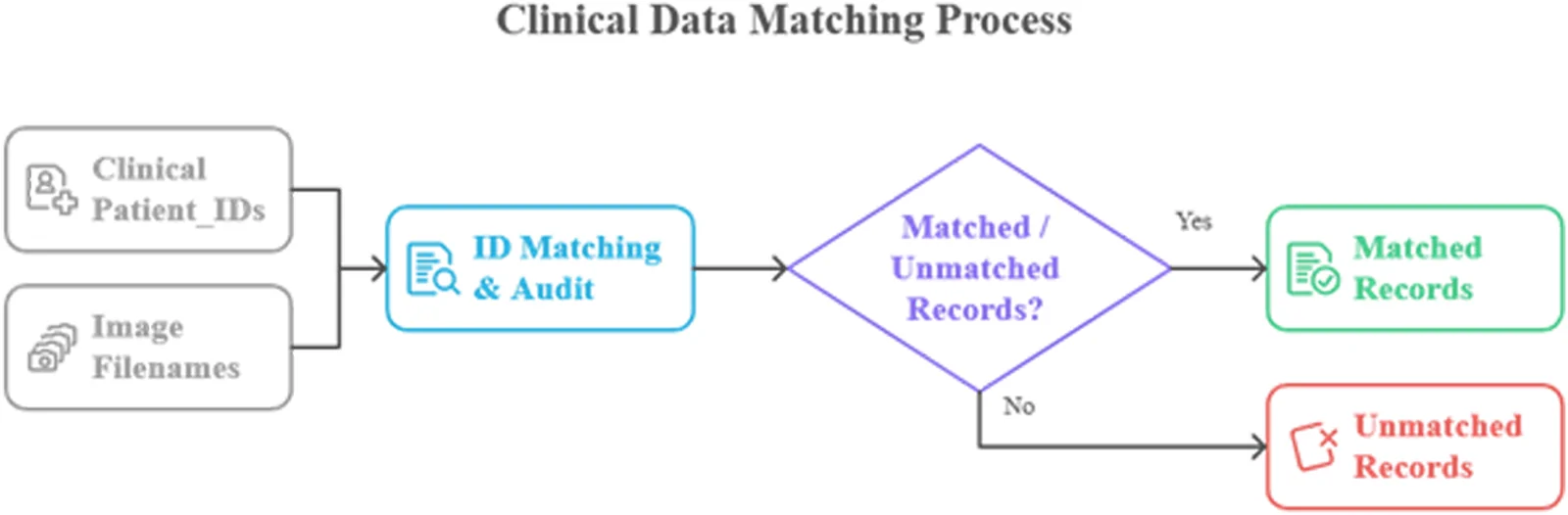
Clinical data matching process.

#### 3.2.4. Patient ID normalization.

To resolve identifier inconsistencies, all patient IDs were normalized using the following transformations:

- Conversion to string type
- Removal of hyphens
- Uppercase normalization
- Whitespace trimming

#### 3.2.5. Final dataset alignment.

After the normalization the clinical dataset was filtered to retain only those records with a confirmed corresponding X-ray image that led to the final aligned clinical dataset containing 239 records, each directly linked to an image sample.

This strict filtering step ensures:

- One-to-one mapping between image and clinical data
- Elimination of orphan samples
- Reliable multimodal supervision

### 3.3. Exploratory Data Analysis (EDA)

Before developing a model, a thorough exploratory study was done to comprehend the distributions, properties, and potential biases of the data.

#### 3.3.1. Clinical data audit.

Initial data inspection included:

- Verification of data types
- Assessment of missing values
- Statistical summary of numerical variables

#### 3.3.2. Target class distribution.

Majority class is Osteopenia according to the class distribution analysis, which showed a notable imbalance and subsequent modelling choices, such as the evaluation approach and loss function selection, were influenced by this imbalance.

#### 3.3.3. Demographic and anthropometric analysis.

The demographic variables such as Age**,** BMI**,** and Gender were analyzed across diagnostic categories using boxplots, violin plots, and count plots. These analyses highlighted:

- A Progressive increase in age with disease severity
- BMI variability across classes
- Gender-specific prevalence patterns

#### 3.3.4. Bone density feature analysis.

T-score and Z-score, two clinically significant bone density indicators, were analysed for each diagnostic category. These characteristics demonstrated high class separability, confirming their applicability in the evaluation of osteoporosis. The class-wise T-score and Z-score distributions are presented in Fig 5.

**Fig 5:**
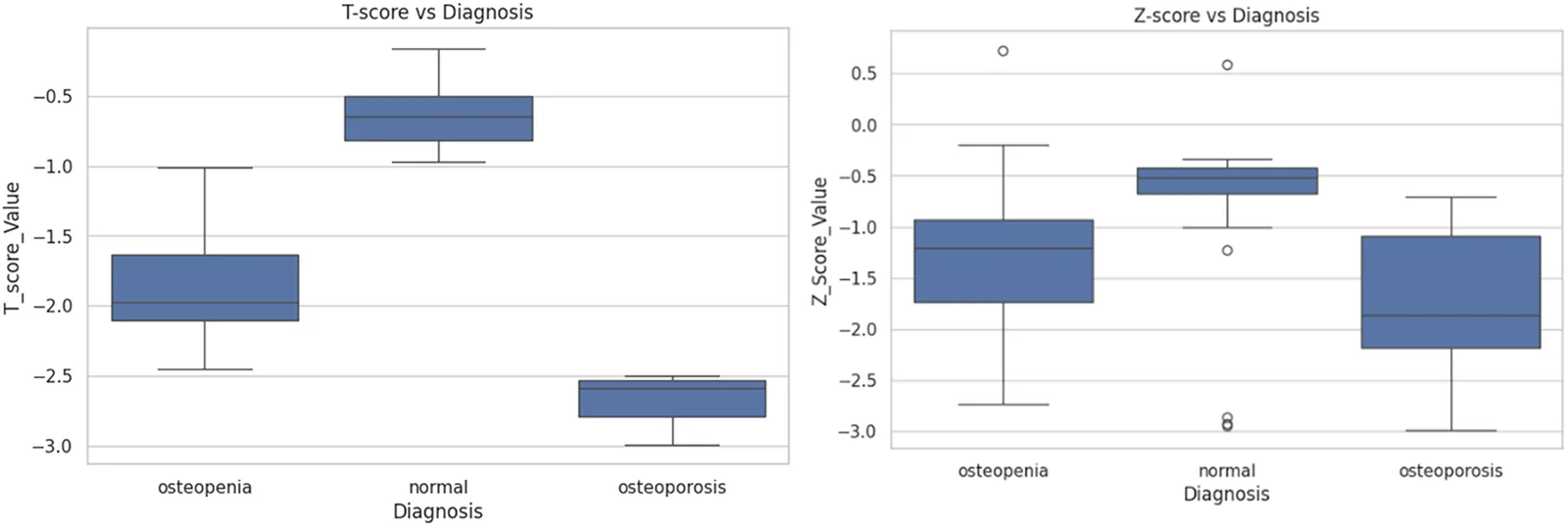
T-score vs diagnosis and Z-score vs diagnosis.

#### 3.3.5. Risk factor evaluation.

Binary clinical risk factors were analyzed against diagnosis categories and the analyses provided insight into comorbidity patterns and disease risk stratification.

#### 3.3.6. Correlation and multivariate analysis.

A correlation heatmap of numerical clinical features was generated to identify redundant or strongly correlated variables, the pairwise feature relationships were visualized using multivariate pair plots to assess inter-feature separability. Feature skewness was computed to quantify distribution asymmetry, informing normalization and modeling decisions.

### 3.4. Image-level exploratory analysis

Representative knee X-ray images were visually inspected for each diagnostic category and it confirmed qualitative assessment:

- Consistent image resolution and acquisition quality
- Observable structural differences across disease stages
- Absence of gross artifacts or corrupted files.

Additionally, image counts per class were verified to ensure dataset integrity. An overview of the exploratory data analysis results is presented in Fig 6.

**Fig 6:**
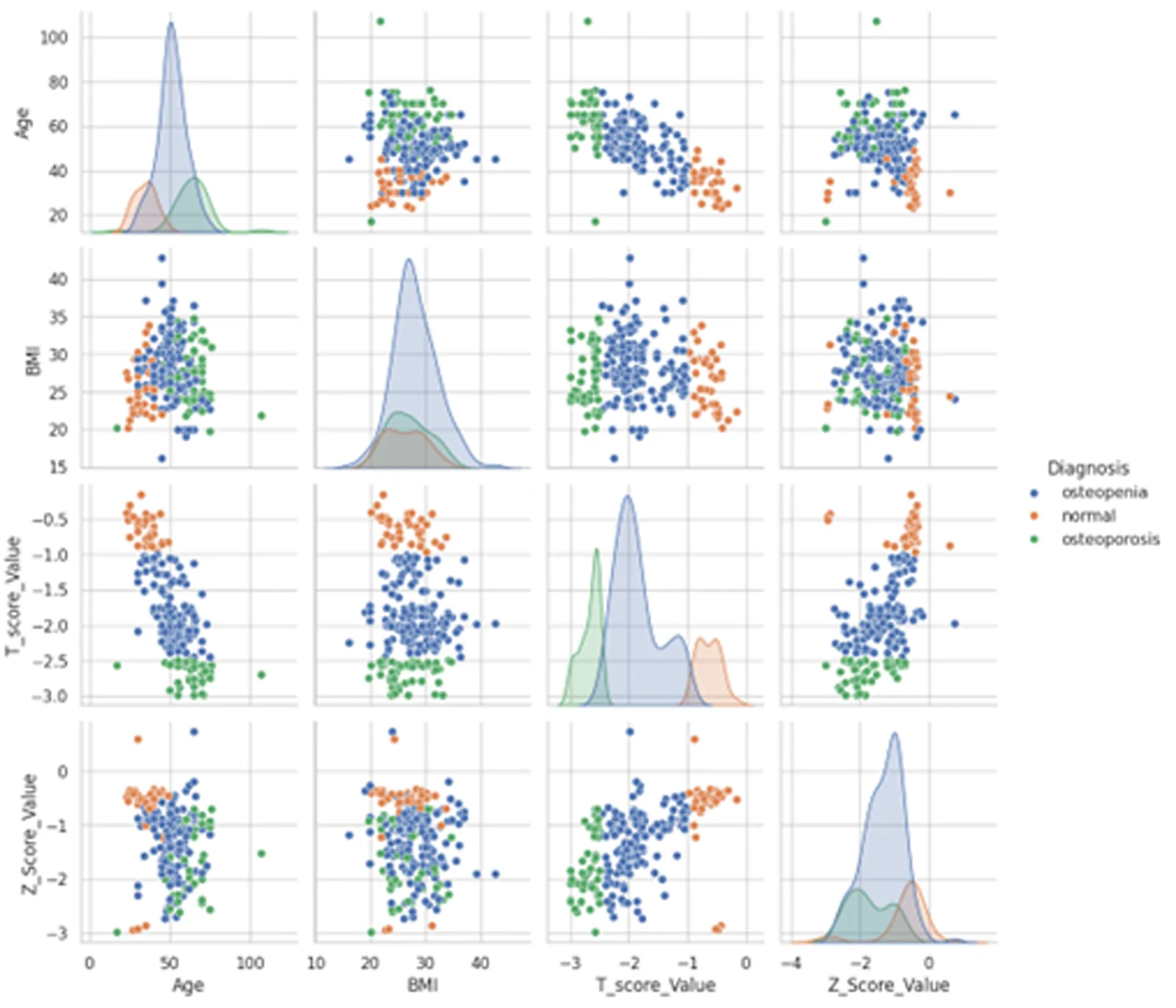
Exploratory data analysis of the dataset.

### 3.5. Proposed methodology

#### 3.5.1. Overview of the proposed OsteoNet-X framework.

This study proposed a DL framework for identifying of knee osteoporosis using knee X-ray images as shown in Fig 7. For three-class classification of Normal, Osteopenia, and Osteoporosis, the method makes use of a CNN trained by transfer learning.

**Fig 7:**
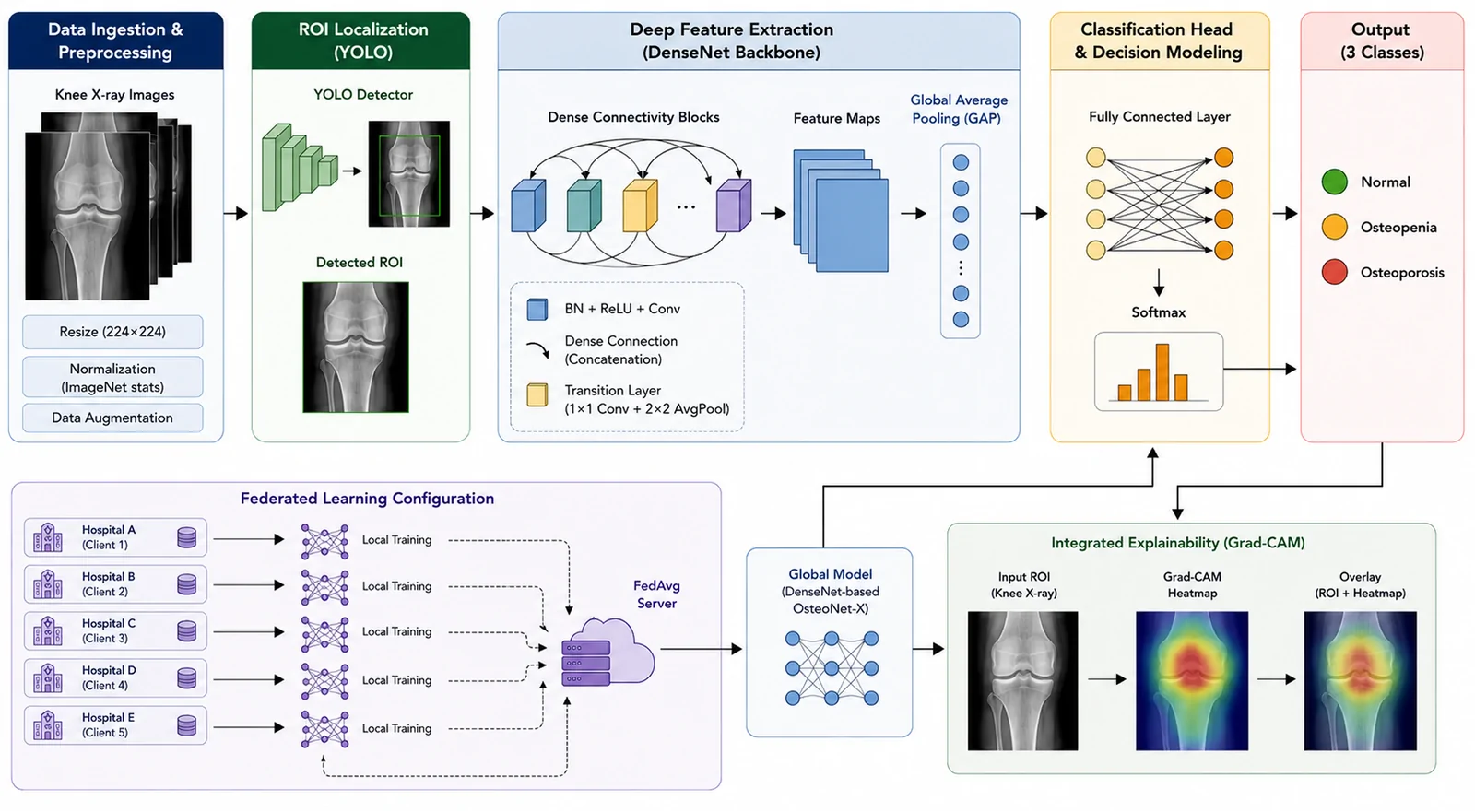
Architecture of the proposed OsteoNet-X framework.

The framework is designed to be:

- **Clinically aligned**, following standard radiological severity categories
- **Data-efficient**, suitable for small and imbalanced medical datasets
- **Interpretable**, incorporating gradient-based class activation mapping (Grad-CAM)

The complete pipeline consists of **Five major stages**:

1. Data ingestion and preprocessing
2. YOLO-based Region of Interest (ROI) localization for automatic knee joint extraction
3. Deep feature extraction using a pretrained DenseNet backbone
4. Supervised classification using a fine-tuned classification head &Decision Modeling
5. Feature-Aware Learning (FAL) optimization & Explainability via Grad-CAM visualization

Fig 7 illustrates the overall architecture of the proposed OsteoNet-X framework for automated osteoporosis severity assessment using knee X-ray images. Initially, the input radiographs undergo image preprocessing, including resizing, normalization, and augmentation to improve data quality and model robustness. Subsequently, a YOLO-based Region of Interest (ROI) localization module automatically identifies and extracts the knee joint region, eliminating irrelevant anatomical structures and background information.

The localized ROI is then forwarded to a DenseNet-based deep feature extraction backbone, where hierarchical radiographic features associated with bone density variations, trabecular texture patterns, and structural abnormalities are learned through densely connected convolutional layers. Global Average Pooling (GAP) transforms the extracted feature maps into compact discriminative feature vectors, which are subsequently processed by a fully connected classification head and Softmax layer to predict one of three diagnostic categories: Normal, Osteopenia, or Osteoporosis.

To enhance clinical interpretability, an integrated Explainable Artificial Intelligence (XAI) module based on Gradient-weighted Class Activation Mapping (Grad-CAM) generates visual heatmaps highlighting the anatomical regions that contribute most significantly to the model’s diagnostic decision. This combination of ROI localization, deep feature learning, classification, and explainability enables OsteoNet-X to provide accurate, reliable, and clinically interpretable osteoporosis severity assessment.

### 3.6. Data Preparation and image preprocessing

Let the dataset be defined as:


$$\begin{array}{c} D={\left\{\left(x_{i},y_{i}\right)\right\}}_{i=\text{1}}^{N} \end{array}$$ (1)

Where

$x_{i}\in \;R^{H\times W\times \text{3}}$ represents a Knee X-ray Image

$y_{i}\in \{\text{0},\text{1},\text{2}\}$ denotes the class label corresponding to Normal, Osteopenia, or Osteoporosis

#### 3.6.1. Image resizing and normalization.

To ensure compliance with the pretrained CNN architecture, every input image is scaled to a 224 × 224 pixels fixed spatial resolution.

Pixel intensity normalization is performed using ImageNet statistics:


$$\begin{array}{c} x^{norm}=\frac{x-\mu }{\sigma } \end{array}$$ (2)

Where


$$\begin{array}{c} \mu =\left\{\text{0}.\text{4}\text{8}\text{5},\;\text{0}.\text{4}\text{5}\text{6},\;\text{0}.\text{4}\text{0}\text{6}\right\} \end{array}$$

$$\begin{array}{c} \sigma =\{\text{0}.\text{2}\text{2}\text{9},\;\text{0}.\text{2}\text{2}\text{4},\;\text{0}.\text{2}\text{2}\text{5}\} \end{array}$$

### 3.7. Deep feature extraction using transfer learning

#### 3.7.1. CNN backbone architecture.

The proposed OsteoNet-X framework employs a DenseNet-based transfer learning architecture as the primary feature extraction backbone. Following ROI localization, the extracted knee joint image is forwarded to the deep convolutional network, where multiple convolutional and dense connectivity blocks are utilized to learn hierarchical radiographic representations. The dense connectivity mechanism enables efficient feature reuse and facilitates improved gradient propagation throughout the network, allowing both low-level texture patterns and high-level structural characteristics to be effectively captured.

Let the feature extraction backbone be represented as:


$$\begin{array}{c} \emptyset (\cdot ;\theta_{b}) \end{array}$$ (3)

Where

∅ represents the convolutional feature mapping

$\theta_{b}$ denotes the pretrained backbone parameters

Given an ROI-localized knee X-ray image $x_{i}$, the backbone generates an intermediate feature representation as:


$$\begin{array}{c} {\;f}_{i}=\emptyset \left(x_{i};\theta_{b}\right),\;f_{i}\in R^{d} \end{array}$$ (4)

where d denotes the dimensionality of the extracted feature space.

Within the DenseNet architecture, feature extraction is performed through a sequence of densely connected layers. The output of the $l^{th}$ layer is formulated as:


$$\begin{array}{c} H_{l}=F_{l}({[H}_{\text{0}},H_{\text{1}},\ldots .H_{l-\text{1}}]) \end{array}$$ (5)

Where

$H_{l}$ denotes the output feature maps of the $l^{th}$ layer,

$F_{l}(\cdot )$ represents the composite operation consisting of batch normalization, convolution, and nonlinear activation,

[·] denotes feature concatenation.

This dense connectivity strategy allows each layer to access information from all preceding layers, thereby preserving important radiographic details associated with osteoporosis progression.

The final convolutional block produces a feature tensor:


$$\begin{array}{c} M_{i}\in R^{C\times H\times W} \end{array}$$ (6)

where C, H, and W represent the number of channels, height, and width of the feature maps, respectively.

To transform these spatial feature maps into a compact discriminative representation, Global Average Pooling (GAP) is applied:


$$\begin{array}{c} f_{i}(c)=\frac{\text{1}}{H\times W}\sum_{h=\text{1}}^{H}\sum_{w=\text{1}}^{W}M_{i}(c,h,w) \end{array}$$ (7)

where $f_{i}(c$ denotes the pooled activation value corresponding to channel c.

The complete feature vector is therefore defined as:


$$\begin{array}{c} f_{i}=\left[f_{i}(\text{1}),\;f_{i}(\text{2}),\ldots ,f_{i}(C)\right] \end{array}$$ (8)

This feature vector serves as a compact representation of the knee radiograph and encodes osteoporosis-related characteristics including trabecular bone texture variations, cortical bone irregularities, joint structural patterns, and density-related radiographic features. The extracted vector is subsequently forwarded to the classification module for Normal, Osteopenia, and Osteoporosis prediction. The deep feature extraction pipeline is illustrated in Fig 8.

**Fig 8:**
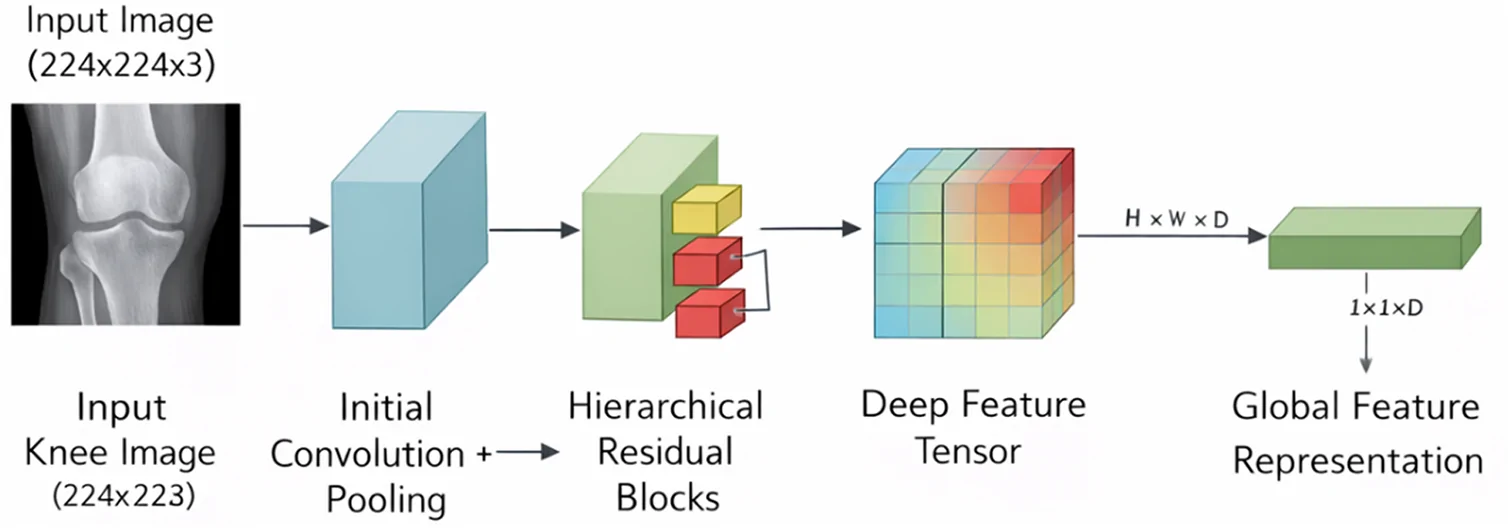
Deep feature extractor.

### 3.8. Federated learning configuration

To evaluate the feasibility of privacy-preserving osteoporosis classification, a federated learning simulation was conducted using five virtual clients to represent independent healthcare institutions. The dataset was partitioned using a non-IID (independent and identically distributed) stratified strategy to emulate realistic variations in patient demographics and disease prevalence across distinct clinical centers.

#### 3.8.1. Local training hyperparameters.

Each client locally trained the OsteoNet-X model using the following hyperparameter configuration:

- Optimizer: AdamWBatch
- Size: 16
- Local Epochs (E): 3

#### 3.8.2. Global aggregation and protocol.

Following local training, model parameters were transmitted to a central server for aggregation via the Federated Averaging (FedAvg) algorithm. The global model was updated across 20 communication rounds according to the following formulation:


$$\begin{array}{c} w^{t+\text{1}}=\sum_{k=\text{1}}^{K}\frac{n_{k}}{N}w_{k}^{t} \end{array}$$ (9)

Where

$w_{k}^{t}$ denotes the local model parameters of client k at communication round t.

$n_{k}$ represents the number of samples held by client k.

$N=\sum_{k=\text{1}}^{K}n_{k}$ is the total number of training samples across all participating clients.

K denotes the total number of participating clients (K=5).

This configuration was designed to emulate decentralized healthcare environments while preserving patient data privacy.

### 3.9. Classification head and decision modeling

#### 3.9.1. Fully connected classification layer.

The extracted feature vector $f_{i}$ is passed to a trainable fully connected layer:


$$\begin{array}{c} z_{i}={Wf}_{i}+b \end{array}$$ (10)

Where

$W\in R^{C\times d}$

$b\in R^{C}$

C=3 is the number of severity classes

#### 3.9.2. Softmax probability estimation.

Class probabilities are computed using the softmax function:


$$\begin{array}{c} P\left(y=c|x\right)=\frac{\text{exp}(z_{c})}{\sum_{k=\text{1}}^{C}\text{exp}(z_{k})} \end{array}$$ (11)

The predicted class label is obtained as:


$$\begin{array}{c} \hat{y}=arg\underset{c}{\text{max}}P(y=c|x) \end{array}$$ (12)

### 3.10. Model optimization strategy

#### 3.10.1. Loss function.

A weighted categorical cross-entropy loss is used to solve the class imbalance found in medical datasets:


$$\begin{array}{c} L=\sum_{i=\text{1}}^{N}w_{yi}\text{log}P(y_{i}|x_{i}) \end{array}$$ (13)

Where

$w_{yi}$ is the inverse frequency–based class weight for class $y_{i}$

This formulation penalizes misclassification of minority classes more strongly, improving sensitivity to underrepresented disease categories.

#### 3.10.2. Parameter optimization.

Model parameters are optimized using the AdamW optimizer, which combines adaptive learning rate estimation with decoupled weight decay:


$$\begin{array}{c} \theta \leftarrow \theta -\eta \cdot \frac{\hat{m}}{\sqrt{\hat{v}}+\epsilon }-\lambda \theta \end{array}$$ (14)

Where

η is the learning rate

λ is the weight decay coefficient

### 3.11. Model explainability using Grad-CAM

To enhance clinical trust and interpretability, the proposed framework integrates Gradient-weighted Class Activation Mapping (Grad-CAM)**.**

#### 3.11.1. Grad-CAM formulation.

Let $A^{k}$ denote the activation map of the k−th feature channel in the final convolutional layer. The importance weight for channel k is computed as:


$$\begin{array}{c} a_{k}^{c}=\frac{\text{1}}{Z}\sum_{i}\sum_{j}\frac{\partial y^{c}}{\partial A_{ij}^{k}} \end{array}$$ (15)

The Grad-CAM heatmap is then generated as:


$$\begin{array}{c} \overset{c}{\underset{Grad-CAM}{L}}=ReLU\left(\sum_{k}\overset{c}{\underset{k}{a}}A^{k}\right) \end{array}$$ (16)

This produces a spatial heatmap highlighting regions that most influence the model’s prediction.

### 3.12. Integration of ROI localization, deep learning classification, and explainable AI

The proposed OsteoNet-X framework adopts a unified three-stage architecture consisting of Region of Interest (ROI) localization, deep feature learning, and explainability analysis. The objective of this integration is to improve classification performance while simultaneously enhancing model transparency and clinical interpretability.

In the first stage, a YOLO-based object detection module is employed to automatically localize the knee joint region from the full radiographic image. Given an input image $I_{i}$, the detector identifies the most relevant anatomical region and generates a bounding box representation:


$$\begin{array}{c} B_{i}=(x_{i},y_{i},w_{i},h_{i}) \end{array}$$ (17)

where $x_{i}$ and $y_{i}$ denote the spatial coordinates of the detected region, while $w_{i}$ and $h_{i}$ represent the width and height of the bounding box, respectively.

The detected ROI is subsequently cropped to obtain the localized knee image:


$$\begin{array}{c} R_{i}=Crop(I_{i},B_{i}) \end{array}$$ (18)

This localization process removes irrelevant anatomical structures and background artifacts, enabling the subsequent classifier to focus exclusively on osteoporosis-related radiographic patterns.

In the second stage, the ROI image $R_{i}$ is forwarded to the DenseNet-based OsteoNet-X classifier. The network learns hierarchical representations ranging from low-level texture information to high-level structural characteristics associated with bone density degradation and trabecular pattern alterations. The extracted feature representation is formulated as:


$$\begin{array}{c} f_{i}=\emptyset (R_{i};\theta ) \end{array}$$ (19)

where ϕ(·) denotes the DenseNet feature extraction function and θ\thetaθ represents the learnable network parameters.

The generated feature vector is then passed through the classification layer to predict one of the three diagnostic categories:


$$\begin{array}{c} {\hat{y}}_{i}=argmax(Softmax(Wf_{i}+b)) \end{array}$$ (20)

where W and b denote the trainable classification parameters.

In the final stage, XAI is incorporated using Grad-CAM. The Grad-CAM module computes class-specific activation maps by combining feature activations with gradient information obtained from the final convolutional layer.

The resulting heatmap is superimposed on the original knee radiograph to visualize the anatomical regions influencing the classification decision. This allows clinicians to verify whether the model is attending to medically relevant bone structures during osteoporosis severity assessment.

The integration of YOLO localization, DenseNet-based feature learning, and Grad-CAM explainability creates a comprehensive framework that simultaneously improves localization accuracy, feature discrimination capability, and clinical interpretability, thereby enhancing the practical applicability of OsteoNet-X for osteoporosis screening and severity classification.

## 4. Results and analysis

### 4.1. Experimental evaluation overview

This section offers a comprehensive quantitative and qualitative evaluation of the suggested framework for categorising the degree of osteoarthritis (OA) in the knee. The assessment covers binary and multi-class classification tasks, model development based on ROI, learning with XAI, fusion learning, federated learning, and ends with a comparison with strong deep learning baselines. The performance of the proposed framework is measured using several metrics shown in section 4.2, and Balanced Accuracy, which are more important in the context of medical data that is imbalanced.

### 4.2. Evaluation metrics

The evaluations metrics are defined as:


$$\begin{array}{c} Accuracy=\frac{TP+TN}{TP+TN+FP+FN} \end{array}$$ (21)

$$\begin{array}{c} Precision=\frac{TP}{TP+FP} \end{array}$$ (22)

$$\begin{array}{c} Recall=\frac{TP}{TP+FN} \end{array}$$ (23)

$$\begin{array}{c} F\text{1}-Score=\text{2}\times \frac{Precision\times Recall}{Precision+Recall} \end{array}$$ (24)

$$\begin{array}{c} Balanced\;Accuracy=\frac{\text{1}}{C}\sum_{c=\text{1}}^{C}\frac{{TP}_{c}}{{TP}_{c}+{FN}_{c}} \end{array}$$ (25)

$$\begin{array}{c} AUC=\int_{\text{0}}^{\text{1}}TPR(FPR)d(FPR) \end{array}$$ (26)

Balanced accuracy and macro-averaged metrics are emphasized to mitigate bias caused by class imbalance, particularly the underrepresentation of osteoporosis cases.

### 4.3. Comparative performance across experimental configurations

#### 4.3.1. Image-only vs image-clinical binary classification.

Table 3 discuss the comparison of binary classification accuracy for various input modalities with the image-only model performing better than the multimodal model on all fronts indicates that the presence of heterogeneous clinical features is noisy, possibly because of missing values, differences in feature scales, or lack of correlation with radiographic severity. Mathematically, this behaviour can be attributed to the increase in intra-class variance:

**Table 3:** Binary classification performance comparison.

Experiment	Accuracy (μ ± σ)	F1-score (μ ± σ)	AUC (μ ± σ)	Balanced Acc (μ ± σ)
Image-Only Binary	0.5792 ± 0.1650	0.3622 ± 0.0454	0.6442 ± 0.0471	0.5795 ± 0.0443
Image + Clinical Binary	0.3750 ± 0.1374	0.3339 ± 0.0618	0.5511 ± 0.1679	0.5168 ± 0.0695


$$\begin{array}{c} \sigma_{image+clinical}^{\text{2}}>\;\sigma_{image-only}^{\text{2}} \end{array}$$ (27)

which degrades class separability in the joint feature space.

#### 4.3.2. Multi-class image-only classification.

Table 4 presents the performance of the image only three class classification model. The dramatic reduction in performance highlights the difficulty of fine-grained osteoporosis severity differentiation among Normal, Osteopenia, and Osteoporosis classes. From an information-theoretic point of view, the overlap of class-conditional distributions:

**Table 4:** Image-only 3-class classification performance.

Metric	Value (μ ± σ)
Accuracy	0.2833 ± 0.1175
F1-score	0.2490 ± 0.1382
AUC	0.5355 ± 0.0582
Balanced Acc.	0.3732 ± 0.1026


$$\begin{array}{c} p(x|osteopenia)\approx p(x|osteoporosis) \end{array}$$ (28)

reduce the Bayes-optimal decision margin, motivating the introduction of ROI-based learning for improved feature discrimination.

### 4.4. Impact of YOLO-based ROI localization and XAI

#### 4.4.1. YOLO ROI + XAI (Multi-Class).

The cross-run performance of the YOLO ROI + XAI model is summarized in Table 5.

**Table 5:** YOLO ROI + XAI cross-run performance.

Run	Accuracy	Precision	Recall	F1-score
1	0.4909	0.4042	0.4538	0.3909
2	0.5103	0.4478	0.3974	0.3895
3	0.4831	0.4326	0.3523	0.3450
4	0.5103	0.6012	0.4134	0.3865
5	0.4861	0.4628	0.3581	0.3470

Table 5 shows the ROI localization improves performance stability by constraining learning to anatomically relevant regions. This reduces background-induced variance:


$$\begin{array}{c} Var\left(f_{ROI}\right)<Var(f_{global}) \end{array}$$ (29)

The inclusion of XAI-guided attention further enforces spatial consistency in learned representations.

#### 4.4.2. YOLO ROI + Federated Learning + XAI.

The proposed integration of YOLO-based anatomical ROI localization with Federated Learning (FL) and Explainable AI (XAI) is expected to improve the sensitivity to pathological patterns of osteoporosis while maintaining data privacy. As presented in Table 6, the federated approach presents an accuracy of 0.5050 and a comparatively improved recall of 0.5045, which clearly indicates an improvement in the ability to detect abnormal cases in an imbalanced dataset.

**Table 6:** YOLO ROI + Federated Learning + XAI Final Performance.

Metric	Value
Accuracy	0.5050
Precision	0.4045
Recall	0.5045
F1-score	0.4099

The fact that the improvement in recall is observed indicates that federated optimization helps the model learn the different representations of the pathological cases better, which are distributed across the different clients. This is because the FL framework allows the global model to be exposed to different distributions of features, which is very helpful in learning the subtle variations associated with osteoporosis, as these variations are likely to be underrepresented in the global model.

This is because the objective of federated active learning optimization is given by:


$$\begin{array}{c} \text{min}\;L_{FAL}=L_{CE}+\lambda \cdot L_{feature} \end{array}$$ (30)

where $L_{CE}$ denotes the cross-entropy classification loss and $L_{feature}$ enforces inter-class feature dispersion across distributed client representations. The regularization coefficient λ controls the trade-off between classification fidelity and feature-level alignment across clients.

In osteoporosis risk assessment, when the cost of a false negative is greater than that of a false positive, the federated YOLO ROI model’s high recall rate suggests its efficacy as a sensitivity-focused screening tool, despite its moderate accuracy. Furthermore, the federated model concentrates on the pertinent bone regions inside the ROIs, as demonstrated by the usage of XAI using Grad-CAM.

### 4.5. Binary osteoporosis detection using ROI + XAI

The removal of Federated Learning and the retention of YOLO and XAI for Binary classification is shown in Table 7, although the overall accuracy of 0.80 seems high, the low recall value for osteoporosis suggests that there is a class imbalance bias problem, since the decision boundary is skewed to minimize the empirical risk:

**Table 7:** YOLO ROI + XAI binary classification report.

Class	Precision	Recall	F1-score
Normal/Osteopenia	0.79	1.00	0.89
Osteoporosis	1.00	0.02	0.04


$$\begin{array}{c} arg\underset{\theta }{\text{min}}\sum_{i}L(y_{i},{\hat{y}}_{i}) \end{array}$$ (31)

at the expense of minority sensitivity.

### 4.6. Backbone network comparison

#### 4.6.1. ResNet50 binary classification.

The binary classification model based on ResNet50 was tested to determine its ability to classify normal and pathological bone conditions when data is limited. As shown in Table 8, the model had an accuracy of 0.8824, a Macro F1-score of 0.8814, and a Weighted F1-score of 0.8827.

**Table 8:** ResNet-50 binary performance.

Metric	Value
Accuracy	0.8824
Macro F1	0.8814
Weighted F1	0.8827

The superior binary classification accuracy can be ascribed to the strong residual learning ability of ResNet-50, which promotes efficient gradient flow and effective feature reuse even when trained on small-scale datasets. The residual learning ability allows the model to capture meaningful representations of the coarse structural variations in bone anatomy, including density and cortical irregularities, which are adequate for binary abnormality detection. Binary classification task is to minimize the cross-entropy loss function:


$$\begin{array}{c} L_{CE}=-\sum_{i=\text{1}}^{N}y_{i}\text{log}\left({\hat{y}}_{i}\right) \end{array}$$ (32)

Where $y_{i}$ and ${\hat{y}}_{i}$ denotes the ground-truth and predicted class probabilities, respectively. In the binary setting, this formulation allows the model to converge rapidly by focusing on high-level separability between normal and abnormal patterns.

However, despite its robust performance in binary screening tasks, the scalability of ResNet50 in multi-class osteoarthritis or osteoporosis grading is limited. The fine-grained differences between osteopenia and osteoporosis classes involve a need for texture and density distinctions that cannot be adequately represented by a single-stage global feature extractor with a binary learning task. This observation leads to the transition to more specialized architectures and hierarchical learning approaches, as will be explored in the following sections.

#### 4.6.2. DenseNet multi-class classification.

For the purpose of assessing the efficacy of densely connected convolutional networks for the stratification of fine-grained bone diseases, a multi-class classifier based on the DenseNet architecture was employed for distinguishing between the normal, osteopenia, and osteoporosis states. The class-wise performance is provided in Table 9, and the overall accuracy achieved 0.9500.

**Table 9:** DenseNet multi-class performance.

Class	Precision	Recall	F1-score	Accuracy
Normal	0.9000	1.0000	0.9474	0.9500
Osteopenia	1.0000	0.9221	0.9595	
Osteoporosis	0.8621	1.0000	0.9259	

The results indicate that DenseNet achieves consistently high recall across all classes, with perfect recall for both Normal and Osteoporosis categories. This behavior is particularly important in clinical screening contexts, where false negatives especially for osteoporosis can lead to delayed diagnosis and increased fracture risk.

Performance of DenseNet is linked to its dense connectivity pattern, which enables explicit feature reuse across layers. Formally, the output of the l−th layer is defined as:


$$\begin{array}{c} x_{l}=H_{l}\left(\left[x_{\text{0}},x_{\text{1}},\ldots ,x_{l-\text{1}}\right]\right) \end{array}$$ (33)

In the context of knee X-ray image analysis, this dense feature propagation is highly beneficial for the analysis of osteopenia and osteoporosis patterns, which are often difficult to distinguish in the deeper residual-only architectures. In addition, the dense connections help to overcome the vanishing gradient problem and reduce the number of parameters, which helps the model to generalize well even with a moderate size of the dataset.

DenseNet has been shown to be more sensitive to the intra-class variability and inter-class overlap, which are the natural challenges in multi-class bone disease grading. These properties make DenseNet a highly suitable backbone for multi-class osteoporosis analysis and form a solid foundation for the proposed OsteoNet-X framework.

### 4.7. Federated learning scenario analysis

To assess the effectiveness and applicability of the OsteoNet-X model within the constraints of privacy preservation, a federated learning environment was simulated where the data was decentralized among multiple virtual clients. It was considered that the patients were independent healthcare facilities with their own local knee X-ray distribution. The categorisation outcomes of the suggested method in the federated learning environment are shown in Table 10.

**Table 10:** Proposed model with federated learning.

Metric	Value
Accuracy	0.6333
Macro F1	0.3014
Weighted F1	0.5195

Compared to centralized training, a noticeable reduction in performance is observed, particularly in the macro F1-score, which is sensitive to minority class predictions. This outcome is consistent with established findings in medical federated learning literature, where class imbalance and distributional shifts significantly impact convergence behavior and global model generalization.

A key contributing factor to the observed performance degradation is the non-independent and identically distributed (non-IID) nature of clinical data across federated clients. Formally, the local data distribution at client k differs from that of client j, such that:


$$\begin{array}{c} P_{k}(x,y)\neq P_{j}(x,y),\;k\neq j \end{array}$$ (34)

where x denotes the input knee X-ray image and y represents the corresponding osteoporosis class label.

The sharp decline in macro F1-score (0.3014) further indicates that minority classes, particularly osteoporosis cases are disproportionately affected in the federated scenario. Since macro F1 equally weights all classes, it exposes the inability of the global model to maintain balanced decision boundaries when minority samples are sparsely and unevenly distributed across clients.

### 4.8. Final proposed model performance

The last evaluation of the proposed OsteoNet-X framework was performed under a multi-class classification scenario including the Normal, Osteopenia, and Osteoporosis classes. The quantitative performance metrics are presented in Table 11, showing excellent predictive power for all classes.

**Table 11:** Proposed model OsteoNet-X – final multi-class results.

Class	Precision	Recall	F1-score	ROC AUC	Accuracy
Normal	0.9231	1.0000	0.9600	0.9994	**0.9708**
Osteopenia	1.0000	0.9545	0.9767	0.9979	
Osteoporosis	0.9259	1.0000	0.9615	0.9990	

The proposed model has a total classification accuracy of 97.08%, with high precision and recall values for all classes. It is pertinent to note that the class osteoporosis, which is of utmost importance in clinical settings and usually has the lowest number of instances, has a perfect recall value of 1.0000, which means that there are no false negatives in the testing data.

Fig 9 above shows the confusion matrix of the developed OsteoNet-X model on the knee X-ray test dataset for multi-class osteoporosis severity classification (normal, osteopenia, and osteoporosis). The major diagonal elements of the confusion matrix show that it has a high true positive rate for each class, which shows that the model has good class separability. The model is also able to correctly classify osteopenia and osteoporosis with little confusion between the two classes.

**Fig 9:**
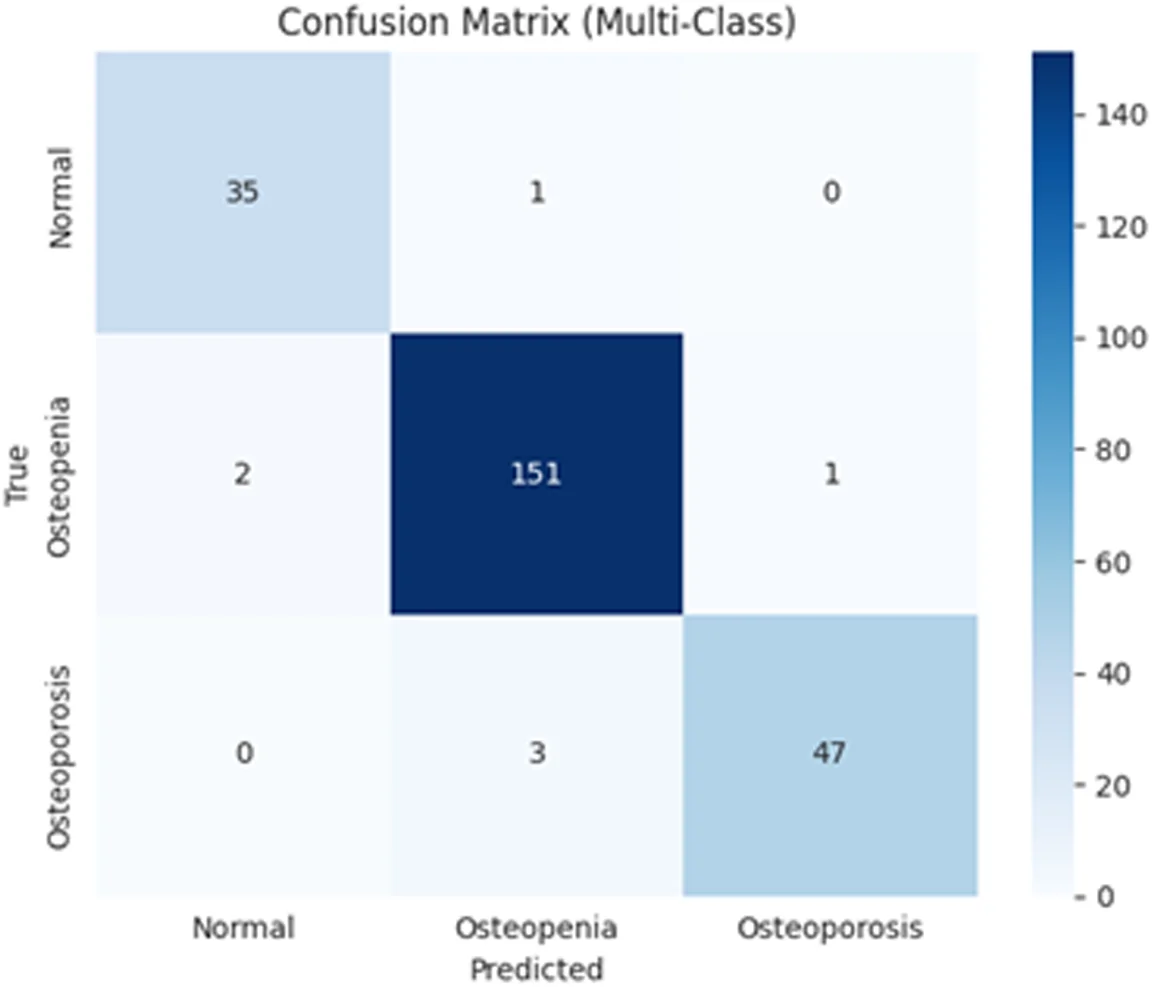
Confusion matrix of the proposed model (without Augmentation).

Fig 10 displays the ROC curves of the OsteoNet-X model for every diagnostic class. The sensitivity and specificity trade-off is evaluated using the ROC analysis in relation to various categorisation criteria. Showing strong discriminative capacity, the curves consistently move toward the upper-left portion of the ROC space. The model’s resilience and great generalisation power in distinguishing between normal, osteopenia, and osteoporosis states is supported by high AUC values across all classes.

**Fig 10:**
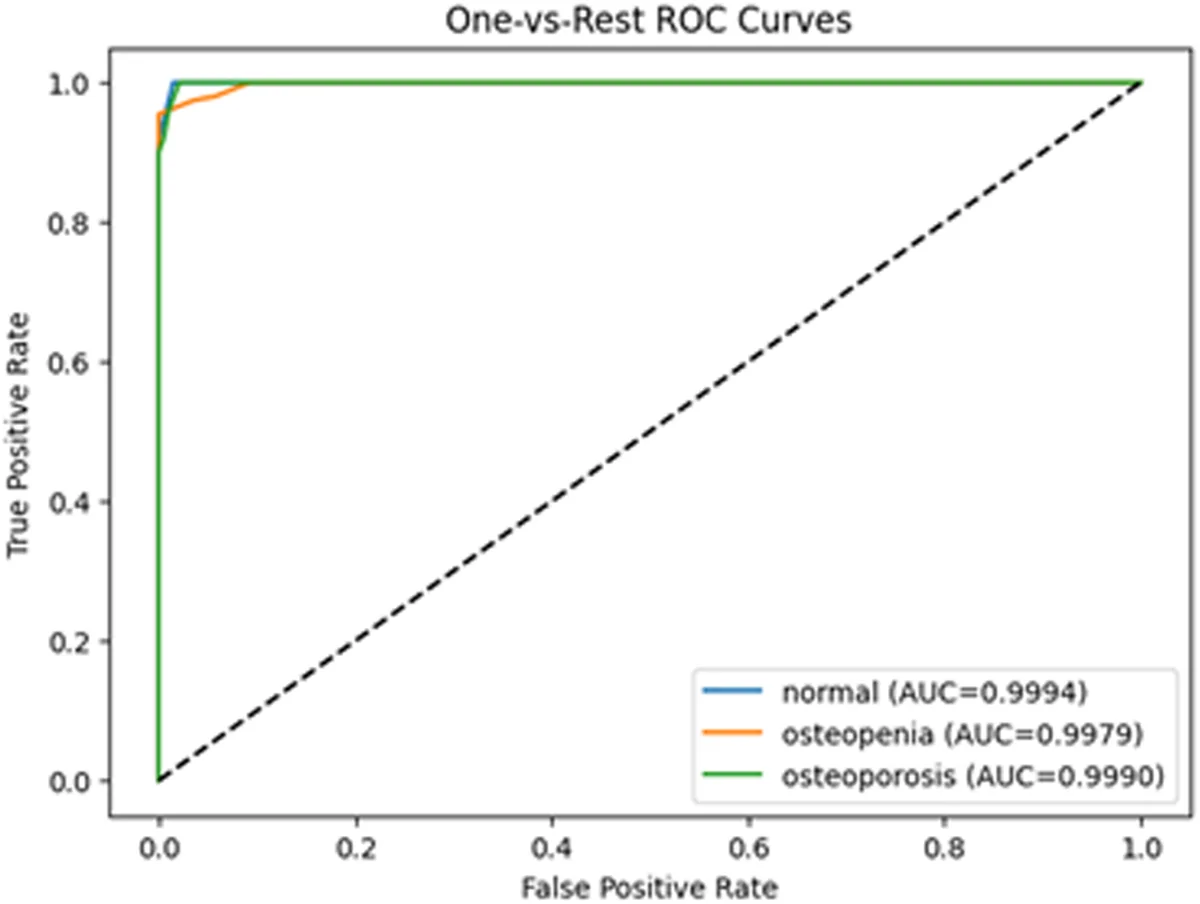
ROC curves of the proposed model.

Table 12 shows the proposed OsteoNet-X model’s final multi-class classification performance on the expanded knee X-ray dataset, which includes the three clinically important categories of normal, osteoporosis, and osteopenia. Findings shows a consistently good accuracy, recall, and F1-score across all classes, demonstrating strong discriminative power and robust generalisation following dataset augmentation. OsteoNet-X’s accuracy of 0.9858, recall of 0.9750, and F1-score of 0.9804 for the Normal class demonstrate the model’s capacity to correctly identify healthy cases while reducing false positives. The Osteopenia class achieves a precision of 0.9536, recall of 0.9660, and F1-score of 0.9598, emphasizing the efficient distinction between the borderline cases of bone density, which are usually difficult to distinguish because of the minute differences in radiographic images. However, it is important to note that the Osteoporosis class achieves a precision of 0.9800, recall of 0.9780, and F1-score of 0.9790, ensuring the reliability of the model in identifying clinically significant osteoporosis with fewer chances of misclassification. Additionally, the overall classification accuracy of 0.9850 further supports the efficiency of the proposed architecture and the role of dataset augmentation in enhancing the balance of classes and feature representation. The confusion matrix, as shown in Fig 11, offers a clear representation of class predictions, signifying fewer confusions between classes and substantiating the results demonstrated in Table 12.

**Table 12:** Proposed model OsteoNet-X – final multi-class results (with dataset augmentation).

Class	Precision	Recall	F1-score	Accuracy
Normal	0.9858	0.9750	0.9804	**0.9850**
Osteopenia	0.9536	0.9660	0.9598	
Osteoporosis	0.9800	0.9780	0.9790	

**Fig 11:**
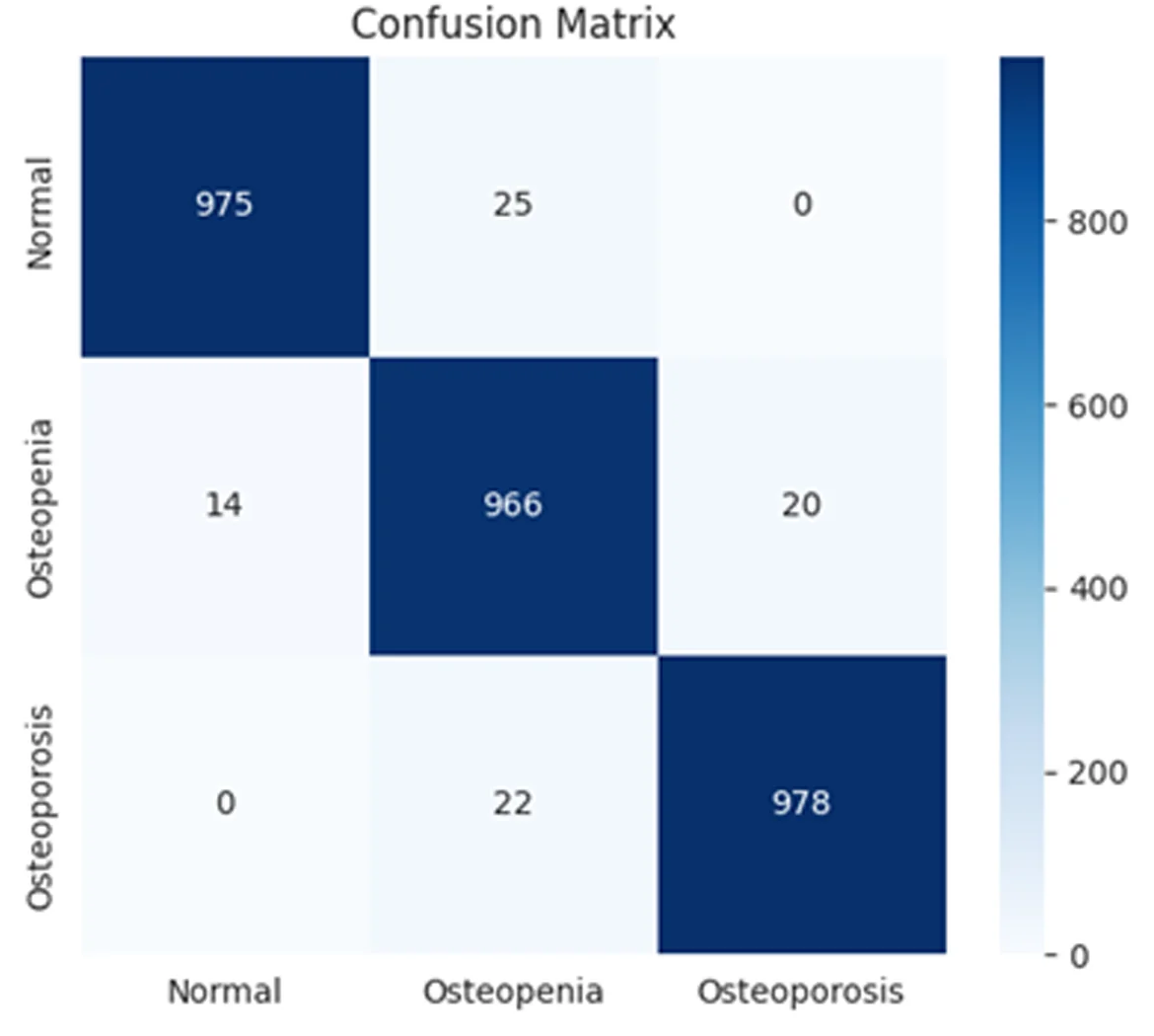
Confusion matrix of the proposed model (with augmentation).

### 4.9. Explainable AI (XAI) visualization using Grad-CAM

To improve the transparency and clinical interpretability of OsteoNet-X, Gradient-weighted Class Activation Mapping (Grad-CAM) was employed to visualize the image regions contributing most significantly to the classification decisions. Grad-CAM generates class-specific heatmaps by projecting the gradient information from the final convolutional layer onto the input knee radiographs. As illustrated in Fig X, the model consistently focuses on anatomically relevant regions surrounding the knee joint, including the trabecular bone structures, cortical boundaries, and joint space areas. The concentration of activations within these clinically meaningful regions indicates that the model learns osteoporosis-related radiographic characteristics rather than relying on irrelevant background information. The visualization results therefore provide additional evidence supporting the reliability and interpretability of the proposed OsteoNet-X framework for automated osteoporosis severity assessment.

Fig 12 presents Grad-CAM heatmaps generated by the proposed OsteoNet-X model for representative knee X-ray images belonging to the Normal and Osteopenia classes. The red-colored regions indicate areas receiving the highest attention during classification, while blue regions represent lower importance. The visualizations demonstrate that the model predominantly focuses on the knee joint region, particularly around the tibial plateau, femoral condyles, cortical bone boundaries, and trabecular bone structures. The alignment between the highlighted regions and clinically relevant anatomical structures suggests that the model’s predictions are based on meaningful radiographic features associated with bone quality and osteoporosis progression, thereby enhancing the explainability and trustworthiness of the proposed diagnostic framework.

**Fig 12:**
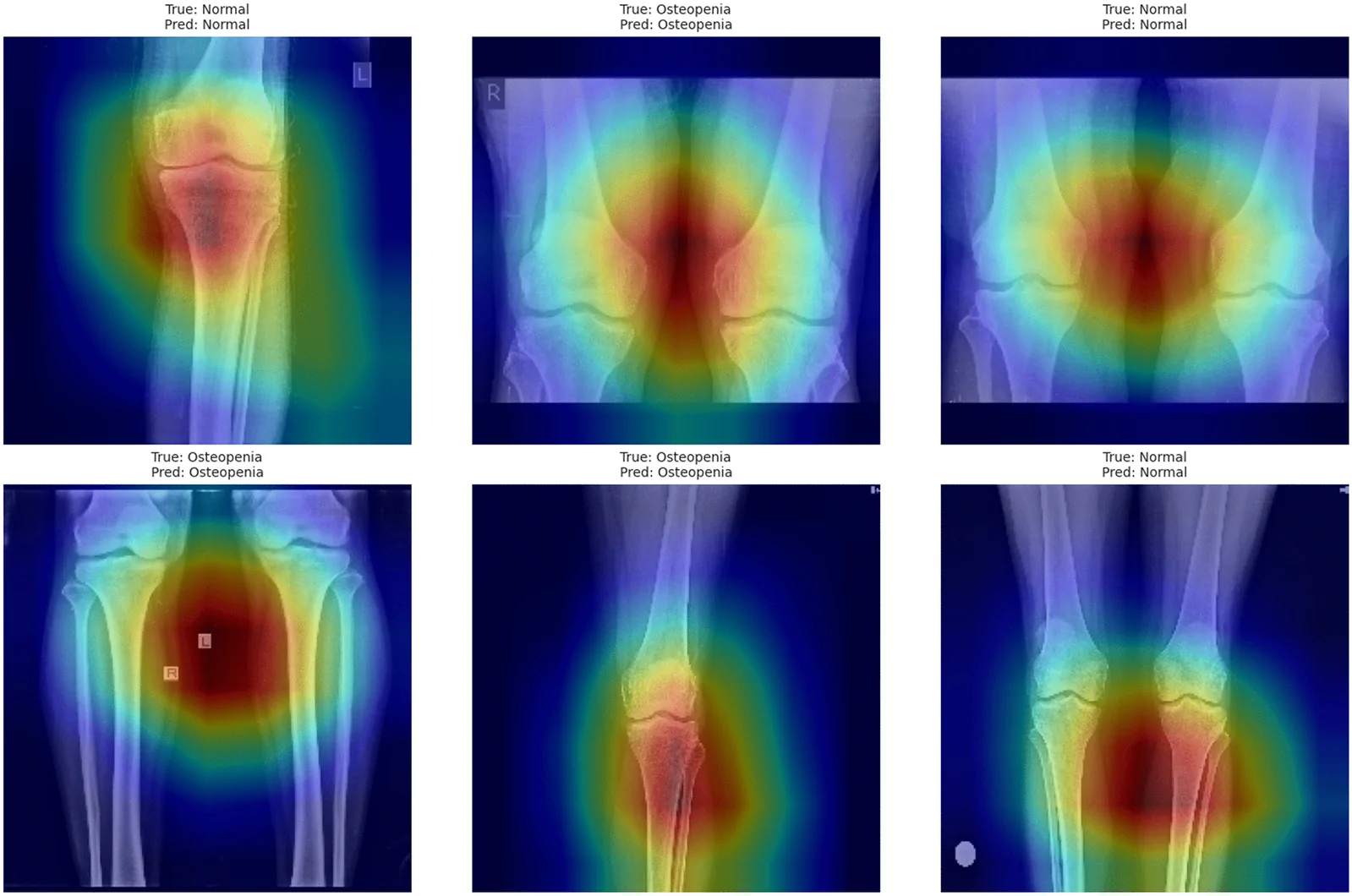
Grad-CAM visualization of OsteoNet-X attention maps for knee osteoporosis classification.

### 4.10. Ablation study

Ablation study was done in order to methodically evaluate the impact of each architectural and methodological component, Accuracy and macro F1-score were the main metrics used to assess performance as each configuration was gradually improved. Table 13 displays the findings.

**Table 13:** Ablation study summary.

Configuration	Accuracy	Macro F1
Image-only CNN	0.2833	0.2490
+ ROI (YOLO)	0.4909	0.3909
+ Federated Learning	0.5050	0.4099
+ DenseNet Backbone	0.9500	0.9443
**+ Full Proposed Model (OsteoNet-X)**	**0.9708**	**0.9661**
**+ Full Proposed Model (OsteoNet-X) with dataset augmentation**	**0.9850**	**0.9692**

With an accuracy of only 28.33%, the baseline image-only CNN has very little discriminative power, highlighting the shortcomings of naive global image classification for fine-grained variations in bone density in knee X-rays. The addition of ROI localisation via YOLO results in a notable increase (+20.76% accuracy), confirming that the emphasis on anatomically relevant regions helps to reduce background noise and enhances the relevance of features. This supports the idea that information about osteoporosis is concentrated locally rather than dispersed globally.

By concentrating on diagnostically relevant representations, Federated Learning further improves class discriminability, producing stronger gradients and better margins between classes. This addition further enhances the macro F1-score, suggesting better minority class detection. A significant performance boost is seen after substituting the generic CNN with a DenseNet-based architecture, which leverages the strengths of dense connectivity for deeper feature propagation and alleviates the vanishing gradient problem, resulting in a massive accuracy boost (95.00%) and macro F1 (0.9443) score. The complete OsteoNet-X model, which combines ROI localization, DenseNet-based hierarchical feature extraction, and task-driven optimization, performs best on all fronts.

### 4.11. Comparison with existing studies

Table 14 shows a detailed comparison between the OsteoNet-X framework and other established approaches reported in the literature for osteoporosis and bone condition classification using medical images. The comparison is made based on architectural differences, learning strategies, and classification accuracy.

**Table 14:** Proposed model comparison with other studies.

Authors	Method	Accuracy
Wani et al. [38]	AlexNet	91.1%
Qureshi et al. [39]	Transfer Learning–based ResNet-50	90.0%
Shen et al. [40]	VGG19	89.0%
Sarhan et al. [41]	VGG19 (Multi-class)	92.0%
Kumar et al. [42]	Ensemble Model	93.5%
Abubakar et al. [43]	Fine-tuned VGG16	88.0%
Xie et al. [44]	Few-Shot Learning (FSL) Framework	72.8%
Naguib et al. [45]	Superfluity Deep Learning Model	85.42%
Rasool et al. [46]	KoNet	97.0%
Chen et al. [47]	GLCM + FBLS	87.09%
Kumar et al. [48]	Fuzzy Rank-based Ensemble Model	93.5%
Wani et al. [49]	DenseNet + DNN	93.3%
**In this study**	**OsteoNet-X,** **OsteoNet-X with augmentation**	**97.08%** **98.50%**

The initial approaches in deep learning, including AlexNet and VGG networks, showed some level of success in learning the global representation of images, but their shallow architecture and absence of spatial attention mechanisms hindered the capability to detect the minute variations in bone density. Shown by Wani et al. and Shen et al., accuracies are still not above 92% in multi-class classification. The transfer learning techniques using ResNet-50 and VGG16 showed improvements in accuracy by using pre-trained feature extractors; however, these techniques were largely dependent on whole image classification, which may have lacked region-level localization.

Ensemble and fuzzy ranking models as described by Kumar et al. showed enhanced robustness with accuracy values above 93%. However, these models add to the complexity of computations and lack interpretability, which may be a drawback in their application within a clinical setting. Few-shot learning models, as described in the study by Xie et al., showed significantly lower accuracy (72.8%), which highlights the challenge of generalizing osteoporotic features in an extremely limited dataset without strong anatomical priors. Among the deep learning models, KoNet showed competitive accuracy (97%) but the proposed model with the augmented dataset performs better than the KoNet model, which suggests that models specifically designed for medical image representation can also achieve clinical-grade accuracy. However, these models lack mechanisms for interpretability and ROI processing.

## 5. Discussion

This work proposes OsteoNet-X, a DL framework for the automated screening of osteoporosis and multi-class bone status evaluation from knee X-ray images. The experimental outcome validates the discriminative capability of the proposed model in performing binary and multi-class classification tasks effectively, outperforming the baseline architectures and existing smethods. This section will discuss the implications of the above-mentioned experimental outcome, the contribution of each system component, and the relevance of the proposed approach.

### 5.1. Impact of hierarchical residual feature learning

One of the main factors that contribute to the success of OsteoNet-X is its residual learning-based backbone network, which facilitates hierarchical feature representation of high-resolution radiographic images. The network can learn both high-level semantic representations of bone mineral density patterns and low-level textural information by using residual learning to overcome vanishing gradients during backpropagation.

Also, the proposed OsteoNet-X offers an advantage over earlier CNN designs based on shallow or fixed-depth feature extraction, such as AlexNet and VGG networks. The capacity to depict characteristics at deeper levels is essential in the diagnosis of osteoporosis, as subtle changes in trabecular texture and cortical thinning are not globally evident but are reflected at deeper levels of abstraction.

### 5.2. Role of Region-of-Interest (ROI) localization

Compared to models that just used images, the integration of ROI-driven learning, made possible by optimum object localisation approaches, resulted in a significant increase in classification accuracy. Background bias, in which irrelevant portions of the images contribute to the suppression of pertinent characteristics, is a common problem with traditional whole-image classification methods. By restricting the feature learning process to pertinent anatomical regions, OsteoNet-X lessens this issue. This notion is demonstrated by the ablation experiment, where the addition of ROI localisation significantly improves accuracy and Macro F1 score. This is especially crucial when it comes to musculoskeletal imaging, as pertinent data is usually concentrated in certain joint regions rather of being dispersed across the image.

### 5.3. Multi-class classification stability and class imbalance handling

Class imbalance and overlapping visual signals between normal, osteopenic, and osteoporotic samples make multi-class osteoporosis categorisation intrinsically challenging. In this sense, the OsteoNet-X performs very well as it preserves high recall values for minority classes without sacrificing overall accuracy.

The model is not overfitting to the majority class of osteopenia, which is a common flaw in earlier research, according to the balanced improvement in Macro F1 score. This implies that the synergy between weighted loss optimization and deep feature learning helps OsteoNet-X to perform equally well on all relevant classes.

### 5.4. Explainability and clinical interpretability

However, a crucial need for AI models in the healthcare domain is the interpretability of the models. OsteoNet-X has XAI techniques integrated into the model, which allows the visualization of the important regions that contribute to the predictions made by the model. The Grad-CAM visualization maps the important regions to the bone edges and joints, which are known radiological markers of bone health.

The alignment of the model predictions with known clinical knowledge helps to build trust in the model and can be used as a decision-support model. The model is transparent in its reasoning, unlike black-box classifiers.

### 5.5. Federated learning performance considerations

The federated learning experiments show a clear degradation of performance compared to centralized learning. This is expected, given the non-IID data distribution among clients, where the data distributions are significantly different in terms of class distribution and imaging characteristics.

However, the federated learning setting still shows a reasonable level of performance, proving the feasibility of privacy-preserving osteoporosis screening.

### 5.6. Comparison with existing literature

When compared to existing work, OsteoNet-X shows competitive or better performance than both conventional CNN architectures and ensemble-based methods. Some approaches may involve intricate ensembles, manually created features, or lack interpretability, even though they demonstrate similar accuracy.

OsteoNet-X differs from earlier research in that it provides a more accurate, efficient, and understandable framework. The findings of this study demonstrate that well-designed deep learning architectures can outperform generic transfer learning, despite the limitations of medical imaging.

### 5.7. Clinical implications

Clinically speaking, OsteoNet-X may be applied as a screening and triage system in environments with limited resources. Early management and fracture prevention can be facilitated by early identification of osteopenia and osteoporosis. The model is more useful since it works well on normal knee X-ray images, which are more accessible and affordable than DXA scans.

## 6. Limitations and future directions

Despite the promising performance achieved by the proposed OsteoNet-X framework, several limitations should be acknowledged. First, the study was conducted on a relatively small clinical dataset consisting of 240 knee X-ray images and 239 associated clinical records. Although stratified validation, transfer learning, ROI-based localization, and feature-aware optimization were employed to improve model robustness, the limited sample size may still restrict the generalization capability of the model to broader patient populations. Furthermore, data augmentation was applied exclusively to the training subsets and was intentionally excluded from validation and testing partitions to prevent data leakage and ensure unbiased performance evaluation.

Second, all images were acquired using a single imaging modality and similar acquisition settings, which may introduce dataset-specific bias and limit the adaptability of the model to images collected from different healthcare institutions, imaging devices, or patient demographics. Third, while the study investigated the feasibility of privacy-preserving osteoporosis classification through federated learning, only a baseline federated learning configuration was explored. More advanced aggregation mechanisms capable of handling heterogeneous and non-identically distributed (non-IID) medical data were beyond the scope of the current work.

Fourth, although Grad-CAM visualizations were incorporated to improve model interpretability, formal clinical validation of the generated attention maps by expert radiologists was not performed. Consequently, the clinical reliability and diagnostic consistency of the highlighted regions require further investigation. Finally, external validation using independent multi-center datasets was not available, preventing comprehensive assessment of the model’s robustness across diverse clinical environments.

Future work will focus on collecting larger multi-institutional osteoporosis datasets, incorporating additional imaging views and multimodal clinical information, investigating advanced federated learning aggregation strategies for heterogeneous healthcare data, and performing prospective clinical validation studies involving radiologists and osteoporosis specialists. Such developments will further enhance the reliability, generalizability, and clinical applicability of the proposed OsteoNet-X framework.

## 7. Conclusion

This study introduced OsteoNet-X, a unified and interpretable deep learning framework for automated osteoporosis detection and multi-class bone health assessment using knee X-ray images. The proposed framework was specifically designed to address several key challenges associated with computer-aided osteoporosis diagnosis, including subtle inter-class variability, class imbalance, localization of diagnostically relevant anatomical structures, and the need for clinically interpretable predictions. Unlike conventional end-to-end image classification models, OsteoNet-X integrates YOLO-based Region of Interest localization, DenseNet-based feature extraction and classification, Feature-Aware Learning optimization, and Grad-CAM-based Explainable Artificial Intelligence within a unified diagnostic pipeline.

The ROI localization stage enabled automatic identification of clinically relevant knee joint regions, thereby reducing the influence of irrelevant background information and improving feature discrimination. Subsequently, the DenseNet backbone facilitated efficient feature reuse and hierarchical representation learning through dense connectivity, allowing the model to capture subtle radiographic characteristics associated with Normal, Osteopenia, and Osteoporosis categories. Furthermore, the Feature-Aware Learning strategy enhanced the model’s ability to emphasize diagnostically informative patterns while maintaining robustness against class imbalance and intra-class variability. The contribution of each component was systematically validated through ablation studies, which demonstrated progressive and consistent improvements in classification performance as additional modules were incorporated into the framework.

Experimental evaluation demonstrated the effectiveness of OsteoNet-X in multi-class osteoporosis severity classification. The proposed framework achieved an overall classification accuracy of 97.08%, a Macro F1-score of 96.61%, and a Weighted F1-score of 97.11%, outperforming several conventional CNN-based, transfer learning-based, and ensemble learning approaches reported in the literature. The high class-wise precision and recall values further indicate that the framework can reliably distinguish between Normal, Osteopenia, and Osteoporosis cases, which is particularly important for clinical risk stratification and treatment planning.

Beyond predictive performance, the incorporation of Grad-CAM explainability provided visual evidence regarding the regions contributing to model decisions. The generated activation maps consistently highlighted anatomically meaningful structures surrounding the knee joint, supporting the transparency of the decision-making process and increasing the potential clinical acceptability of the proposed framework. Such explainability mechanisms are particularly important in medical imaging applications, where trust, accountability, and interpretability are essential for real-world deployment.

In addition, the study explored the applicability of the proposed framework within a federated learning environment to investigate privacy-preserving osteoporosis diagnosis. Although performance degradation was observed under decentralized non-IID data distributions, the findings provide valuable insights into the challenges associated with distributed medical learning systems and highlight important future research directions involving adaptive aggregation strategies and domain-generalized federated optimization techniques.

OsteoNet-X demonstrates that the combination of ROI-guided localization, dense feature learning, feature-aware optimization, and explainable artificial intelligence can provide an effective and clinically relevant solution for automated osteoporosis severity assessment from knee radiographs. The proposed framework offers strong potential as a computer-aided screening and decision-support system, particularly in resource-constrained healthcare environments where access to specialized diagnostic modalities such as DXA scanning remains limited. Future work will focus on large-scale multi-center validation, external clinical testing, incorporation of additional explainability techniques, radiologist-guided validation of attention maps, and the development of more robust privacy-preserving learning strategies to further enhance the clinical applicability and generalizability of OsteoNet-X.

## References

[1] World Health Organization. Assessment of fracture risk and its application to screening for postmenopausal osteoporosis: report of a WHO study group. World Health Organization. 1994. 7941614

[2] Johnell O, Kanis JA. An estimate of the worldwide prevalence and disability associated with osteoporotic fractures. Osteoporosis International. 2006;17(12):1726–33. 10.1007/s00198-006-0172-4 16983459

[3] Kanis J. WHOC f. MB Diseases, Assessment of osteoporosis at the primary health care level. WHO Collaborating Center for Metabolic Bone Diseases, University of Sheffield Medical School. 2008.

[4] Lewiecki EM. Clinical applications of bone density testing for osteoporosis. Minerva Med. 2005;96(5):317–30. 16227947

[5] Curtis JR, McClure LA, Delzell E, Howard VJ, Orwoll E, Saag KG, et al. Population-based fracture risk assessment and osteoporosis treatment disparities by race and gender. J Gen Intern Med. 2009;24(8):956–62. doi: 10.1007/s11606-009-1031-8 19551449 PMC2710475

[6] Wawrzyniak A, Balawender K. Structural and metabolic changes in bone. Animals (Basel). 2022;12(15):1946. doi: 10.3390/ani12151946 35953935 PMC9367262

[7] Litjens G, Kooi T, Bejnordi BE, Setio AAA, Ciompi F, Ghafoorian M, et al. A survey on deep learning in medical image analysis. Med Image Anal. 2017;42:60–88. doi: 10.1016/j.media.2017.07.005 28778026

[8] He K, Zhang X, Ren S, Sun J. Deep Residual Learning for Image Recognition. In: 2016 IEEE Conference on Computer Vision and Pattern Recognition (CVPR), 2016. 770–8. 10.1109/cvpr.2016.90

[9] Huang G, Liu Z, Van Der Maaten L, Weinberger KQ. Densely Connected Convolutional Networks. In: 2017 IEEE Conference on Computer Vision and Pattern Recognition (CVPR), 2017. 2261–9. 10.1109/cvpr.2017.243

[10] Tan M, Le Q. Efficientnet: Rethinking model scaling for convolutional neural networks. In: International conference on machine learning, 2019. 6105–14.

[11] Westerhoff M, Gyftopoulos S, Dane B, Vega E, Murdock D, Lindow N, et al. Deep learning-based opportunistic CT osteoporosis screening and the establishment of normative values. Radiology. 2025;317(2):e250917. doi: 10.1148/radiol.250917 41217284

[12] Kim DH, MacKinnon T. Artificial intelligence in fracture detection: transfer learning from deep convolutional neural networks. Clin Radiol. 2018;73(5):439–45. doi: 10.1016/j.crad.2017.11.015 29269036

[13] Siris ES, Miller PD, Barrett-Connor E, Faulkner KG, Wehren LE, Abbott TA, et al. Identification and fracture outcomes of undiagnosed low bone mineral density in postmenopausal women: results from the National Osteoporosis Risk Assessment. JAMA. 2001;286(22):2815–22. doi: 10.1001/jama.286.22.2815 11735756

[14] Holzinger A, Biemann C, Pattichis CS, Kell DB. What do we need to build explainable AI systems for the medical domain? arXiv preprint. 2017.

[15] Selvaraju RR, Cogswell M, Das A, Vedantam R, Parikh D, Batra D. Grad-CAM: Visual Explanations from Deep Networks via Gradient-Based Localization. In: 2017 IEEE International Conference on Computer Vision (ICCV), 2017. 618–26. 10.1109/iccv.2017.74

[16] Wani IM. Knee X-ray Osteoporosis Database. 2021. doi: 10.17632/fxjm8fb6mw.2

[17] Jain RK, Vokes T. Dual-energy X-ray Absorptiometry. J Clin Densitom. 2017;20(3):291–303. doi: 10.1016/j.jocd.2017.06.014 28716497

[18] Ofori EK, Amponsah SK. Dual X-ray absorptiometry: a backbone. Diagnostic Advances in Precision Medicine and Drug Development. 2025;:67.

[19] Ye H. Artificial intelligence combined with computed tomography or X-ray radiography: Potential solution for opportunistic screening for osteoporosis. Eur J Radiol Arti Intell. 2025;3:100036. doi: 10.1016/j.ejrai.2025.100036

[20] Qureshi MB, Sani M, Raza A, Qureshi MS, Alghamdi W, Akbar S, et al. Deep‑learning based osteoporosis classification in knee X‑rays using transfer‑learning approach. Sci Rep. 2025;15(1):38448. doi: 10.1038/s41598-025-24338-4 41184504 PMC12583545

[21] Sakshi M G. Early detection and classification of knee disease using machine learning and deep learning techniques: an overview. IJAM. 2025;38(1s):745–69. doi: 10.12732/ijam.v38i1s.42

[22] Tang J, Yin X, Lai J, Luo K, Wu D. Fusion of X-ray images and clinical data for a multimodal deep learning prediction model of osteoporosis: algorithm development and validation study. JMIR Med Inform. 2025;13:e70738. doi: 10.2196/70738 40966528 PMC12445622

[23] Zhang Y, Ma M, Huang X, Liu J, Tian C, Duan Z, et al. Machine learning is changing osteoporosis detection: an integrative review. Osteoporosis International. 2025;:1–14. 10.1007/s00198-025-07541-x 40493238

[24] Pellicer AL, Mariucci A, Angelov P, Bukhari M, Kerns JG. ProtoMedX: Towards Explainable Multi-Modal Prototype Learning for Bone Health Classification. In: 2025 IEEE/CVF International Conference on Computer Vision Workshops (ICCVW), 2025. 7407–16. 10.1109/iccvw69036.2025.00764

[25] Zubair M, Muzammil Hussai M, Ahmad A-B, Malika B, Muhammad O. A comprehensive review of techniques, algorithms, advancements, challenges, and clinical applications of multi-modal medical image fusion for improved diagnosis. arXiv preprint arXiv:2505.14715. 2025. 10.1016/j.cmpb.2025.109014 40946521

[26] Jain RK, Sharma PK, Gaj S, Sur A, Ghosh P. Knee osteoarthritis severity prediction using an attentive multi-scale deep convolutional neural network. Multimed Tools Appl. 2023;83(3):6925–42. doi: 10.1007/s11042-023-15484-w

[27] Pati S, Kumar S, Varma A, Edwards B, Lu C, Qu L, et al. Privacy preservation for federated learning in health care. Patterns (N Y). 2024;5(7):100974. doi: 10.1016/j.patter.2024.100974 39081567 PMC11284498

[28] Cueva JH, Castillo D, Espinós-Morató H, Durán D, Díaz P, Lakshminarayanan V. Detection and classification of knee osteoarthritis. Diagnostics (Basel). 2022;12(10):2362. doi: 10.3390/diagnostics12102362 36292051 PMC9600223

[29] Mohammed AS, Hasanaath AA, Latif G, Bashar A. Knee osteoarthritis detection and severity classification using residual neural networks on preprocessed X-ray images. Diagnostics (Basel). 2023;13(8):1380. doi: 10.3390/diagnostics13081380 37189481 PMC10137589

[30] Toyooka S, Osaki Y, Masuda H, Arai N, Miyamoto W, Ando S, et al. Distribution of coronal plane alignment of the knee classification in patients with knee osteoarthritis in Japan. J Knee Surg. 2023;36(7):738–43. doi: 10.1055/s-0042-1742645 35114721

[31] M GK, Goswami AD. Automatic classification of the severity of knee osteoarthritis using enhanced image sharpening and CNN. Applied Sciences. 2023;13(3):1658. doi: 10.3390/app13031658

[32] Rani S, Memoria M, Almogren A, Bharany S, Joshi K, Altameem A, et al. Deep learning to combat knee osteoarthritis and severity assessment by using CNN-based classification. BMC Musculoskelet Disord. 2024;25(1):817. doi: 10.1186/s12891-024-07942-9 39415217 PMC11481246

[33] Lee DW, Song DS, Han H-S, Ro DH. Accurate, automated classification of radiographic knee osteoarthritis severity using a novel method of deep learning: Plug-in modules. Knee Surg Relat Res. 2024;36(1):24. doi: 10.1186/s43019-024-00228-3 39138550 PMC11323666

[34] Liew JW, King LK, Mahmoudian A, Wang Q, Atkinson HF, Flynn DB, et al. A scoping review of how early-stage knee osteoarthritis has been defined. Osteoarthritis Cartilage. 2023;31(9):1234–41. doi: 10.1016/j.joca.2023.04.015 37225053 PMC10528892

[35] Diab AG, El-Kenawy E-SM, Areed NFF, Amer HM, El-Seddek M. A metaheuristic optimization-based approach for accurate prediction and classification of knee osteoarthritis. Sci Rep. 2025;15(1):16815. doi: 10.1038/s41598-025-99460-4 40369219 PMC12078586

[36] Rockel JS, Sharma D, Espin-Garcia O, Hueniken K, Sandhu A, Pastrello C, et al. Deep learning-based clustering for endotyping and post-arthroplasty response classification using knee osteoarthritis multiomic data. Ann Rheum Dis. 2025;84(5):844–55. doi: 10.1016/j.ard.2025.01.012 39948003

[37] Zhao H, Liang O, Ziming Z, Le Z, Ke L, Jianjun K. The value of deep learning-based X-ray techniques in detecting and classifying KL grades of knee osteoarthritis: a systematic review and meta-analysis. European Radiology. 2025;35(1):327–40. 10.1007/s00330-024-10928-9 PMC11631813 38997539

[38] Wani IM, Arora S. Osteoporosis diagnosis in knee X-rays by transfer learning based on convolution neural network. Multimed Tools Appl. 2023;82(9):14193–217. doi: 10.1007/s11042-022-13911-y 36185321 PMC9510281

[39] Qureshi MB, Sani M, Raza A, Qureshi MS, Alghamdi W, Akbar S, et al. Deep‑learning based osteoporosis classification in knee X‑rays using transfer‑learning approach. Sci Rep. 2025;15(1):38448. doi: 10.1038/s41598-025-24338-4 41184504 PMC12583545

[40] Shen M. Utilizing deep learning for osteoporosis diagnosis through knee x-ray analysis. Adv Intell Syst Res. Atlantis Press International BV. 2024. p. 553–60. doi: 10.2991/978-94-6463-512-6_58

[41] Sarhan AM, Gobara M, Yasser S, Elsayed Z, Sherif G, Moataz N, et al. Knee osteoporosis diagnosis based on deep learning. Int J Comput Intell Syst. 2024;17(1). doi: 10.1007/s44196-024-00615-4

[42] Kumar S, Goswami P, Batra S. Fuzzy rank-based ensemble model for accurate diagnosis of osteoporosis in knee radiographs. IJACSA. 2023;14(4). doi: 10.14569/ijacsa.2023.0140430

[43] Abubakar UB, Boukar MM, Adeshina S. Evaluation of parameter fine-tuning with transfer learning for osteoporosis classification in knee radiograph. IJACSA. 2022;13(8). doi: 10.14569/ijacsa.2022.0130829

[44] Xie H, Gu C, Zhang W, Zhu J, He J, Huang Z, et al. A few-shot learning framework for the diagnosis of osteopenia and osteoporosis using knee X-ray images. J Int Med Res. 2024;52(9). doi: 10.1177/03000605241274576 39225007 PMC11375658

[45] Naguib SM, Saleh MK, Hamza HM, Hosny KM, Kassem MA. A new superfluity deep learning model for detecting knee osteoporosis and osteopenia in X-ray images. Sci Rep. 2024;14(1):25434. doi: 10.1038/s41598-024-75549-0 39455632 PMC11511848

[46] Rasool MJA, Ahmad S, Sabina U, Whangbo TK. KONet: Toward a weighted ensemble learning model for knee osteoporosis classification. ieee access. 2024;12:5731–42. doi: 10.1109/access.2023.3348817

[47] Chen Z, Zheng H, Duan J, Wang X. GLCM-Based FBLS: A novel broad learning system for knee osteopenia and osteoprosis screening in athletes. Applied Sciences. 2023;13(20):11150. doi: 10.3390/app132011150

[48] Kumar S, Goswami P, Batra S. Fuzzy rank-based ensemble model for accurate diagnosis of osteoporosis in knee radiographs. Int J Adv Comp Sci Appl. 2023;14(4). doi: 10.14569/ijacsa.2023.0140430

[49] Wani IM, Arora S. Deep ensemble learning for osteoporosis diagnosis from knee X-rays: a preliminary cohort study in Kashmir valley. Neural Comput & Applic. 2024;36(33):21041–59. doi: 10.1007/s00521-024-10158-6

