# Supplementary material for: OsteoNet-X: A deep residual learning framework for automated osteoporosis detection from knee X-ray images
Source: PLoS One. 2026 Oct 27;21(10):e0361033. doi: 10.1371/journal.pone.0361033 (PMC13712691; doi:10.1371/journal.pone.0361033)
Supplement: Supplementary file 1 [file pone.0361033.s002.docx]

**Response to Reviewer Comments:**

We sincerely thank the Editor and reviewers for their comprehensive and insightful comments. We have carefully addressed each point raised, and the manuscript has been substantially revised to reflect these valuable suggestions.

**Reviewer #1:**

**Comment:**

1-The paper described two different models without clearly distinguishing them. The title and abstract described OsteoNet-X as a "deep residual learning framework" trained end-to-end, without ROI extraction or multimodal fusion. But the ablation study in Table 13 showed a completely different incremental pipeline: baseline CNN → YOLO ROI → Federated Learning → DenseNet backbone → Full OsteoNet-X. The full model in that table used YOLO localization and DenseNet, not a standalone ResNet. The introduction and methodology described one architecture; the results reported another. This inconsistency was never resolved in the paper, and it made it genuinely difficult to understand what OsteoNet-X actually was.

**Response:**

We sincerely thank the reviewer for identifying this important issue regarding the architectural consistency of the proposed framework. We acknowledge that the original manuscript did not sufficiently distinguish between the baseline residual learning architecture and the enhanced OsteoNet-X framework utilized in the final experiments.

To address this concern, the manuscript has been extensively revised to clearly define OsteoNet-X as the final integrated framework composed of the following sequential components:

1. YOLO-based Region of Interest (ROI) localization for automatic knee joint extraction,
2. DenseNet-based deep feature extraction and classification,
3. Feature-Aware Learning (FAL) optimization,
4. Explainable AI (XAI) using Grad-CAM visualization.

The earlier ResNet-based architecture is now explicitly described as the baseline deep residual learning model used during preliminary experimentation and comparative evaluation. It is no longer presented as the final architecture of OsteoNet-X.

**Comment:**
2-The dataset was critically small for the claims being made. The study used 240 knee X-ray images across three classes, with a test set inferred to be around 240 images for the without-augmentation condition (from the confusion matrix totals: 36 Normal, 154 Osteopenia, 50 Osteoporosis). Reporting 97.08% accuracy on a test set of this size — roughly 240 samples — did not constitute strong evidence of generalization. A model that memorized coarse class-level features could plausibly reach those numbers. The augmented dataset confusion matrix showed roughly 2,000 test images, suggesting augmentation substantially increased the effective test set size, but the augmentation protocol itself was not described: what transformations were applied, at what multiplicity, and whether augmented images were present in both training and test splits. If augmented images from the same original samples appeared in both, test performance would be inflated.

**Response:**

We sincerely thank the reviewer for this important observation regarding the limited dataset size and the potential risk of overfitting. We acknowledge that the study was conducted using a clinically validated dataset consisting of 240 knee X-ray images, which is relatively small compared with large-scale public medical imaging repositories. However, the primary objective of this work was to investigate the feasibility of automated osteoporosis severity classification using a real-world clinical dataset characterized by inherent class imbalance and limited sample availability, conditions that are commonly encountered in medical imaging research.

To improve model robustness and minimize overfitting, several mitigation strategies were incorporated into the proposed framework. First, stratified cross-validation was employed to ensure balanced class representation across training and validation partitions. Second, transfer learning techniques utilizing pretrained deep convolutional networks were adopted to leverage knowledge learned from large-scale image datasets. Third, adaptive optimization and regularization mechanisms were employed during training to improve model generalization. Furthermore, YOLO-based region-of-interest (ROI) localization was introduced to focus the learning process on diagnostically relevant knee joint regions while reducing background redundancy and irrelevant image information. In addition, the Feature-Aware Learning (FAL) strategy was incorporated to enhance discriminative feature extraction and improve classification stability.

We would also like to clarify that data augmentation was performed exclusively on the training subsets and was never applied to the validation or testing partitions. This precaution was implemented to prevent data leakage and ensure an unbiased evaluation of model performance. The augmentation pipeline consisted of random horizontal flipping, small-angle rotations, contrast enhancement, brightness normalization, and random affine transformations. Detailed descriptions of the augmentation procedures, augmentation factors, and data partitioning strategy have now been included in the revised manuscript to improve transparency and reproducibility.

We fully agree that external validation using larger multi-center datasets would provide stronger evidence regarding model generalizability and clinical applicability. Accordingly, this limitation has now been explicitly acknowledged in the Discussion section, and future work has been proposed to evaluate OsteoNet-X on larger and more diverse clinical cohorts collected from multiple healthcare institutions.

**Comment:**
3-The binary classification results were substantially lower than the multi-class results, which was not explained. The image-only binary classification achieved an accuracy of only 57.92% (Table 3), while the final multi-class model achieved 97.08%. This was a counterintuitive finding — binary tasks are generally easier — and the paper attributed it to multimodal noise without providing evidence. No binary classification result using the same final architecture (DenseNet + ROI) was reported, making it impossible to assess whether the performance gap reflected the architecture or the task.

**Response:**

We thank the reviewer for this insightful observation. The lower binary classification performance observed in the earlier experiments corresponded to preliminary image-only baseline models that did not incorporate ROI localization, DenseNet optimization, or feature-aware learning. These experiments were intentionally included to demonstrate the progressive contribution of each architectural component through ablation analysis.

The final OsteoNet-X framework was optimized specifically for the multi-class osteoporosis severity classification problem, where inter-class structural variations become more discriminative after ROI localization and DenseNet feature extraction.

To clarify this issue, the revised manuscript now explicitly distinguishes:

- Baseline binary experiments,
- Intermediate ROI-based experiments,

Additional explanation has also been added to discuss how class imbalance and overlapping radiographic characteristics between normal and osteopenia samples negatively affected the earlier binary baselines. Furthermore, we now explicitly state that the binary experiments were exploratory baselines rather than the finalized optimized framework.

**Comment:**

4-The federated learning simulation was not described with enough detail to be reproducible. The paper stated that data was "decentralized among multiple virtual clients" simulating independent healthcare facilities, but the number of clients, the data partitioning strategy, the aggregation algorithm (FedAvg or otherwise), the number of communication rounds, and the local training epochs were not specified. Without these details, the federated learning results in Table 10 could not be replicated or compared against established baselines.

**Response:**

We sincerely thank the reviewer for emphasizing the importance of reproducibility in the federated learning (FL) simulation. We agree that the original manuscript did not provide sufficient implementation details regarding the decentralized training configuration.

To address this concern, the Federated Learning subsection has been substantially revised to include all experimental parameters required for reproducibility. Specifically, the FL simulation was conducted using five virtual clients, representing independent healthcare institutions with heterogeneous patient populations. To emulate realistic clinical environments, a non-identically distributed (non-IID) stratified data partitioning strategy was adopted, ensuring that each client possessed different class distributions while preserving the overall diagnostic categories.

Model aggregation was performed using the widely adopted Federated Averaging (FedAvg) algorithm. The federated training process consisted of 20 communication rounds, with each client performing three local training epochs before transmitting model updates to the central aggregation server. The AdamW optimizer was utilized for local optimization with a batch size of 16 samples per client.

It is important to note that the federated learning experiment was included as a supplementary robustness and privacy-preservation evaluation rather than the primary optimization mechanism of OsteoNet-X. The objective was to investigate the feasibility of deploying the proposed framework within decentralized healthcare environments where direct sharing of patient data may not be permissible. The observed performance degradation under federated learning conditions was primarily attributed to the heterogeneous non-IID distributions across participating clients, which is a well-recognized challenge in medical federated learning systems.

The manuscript has now been updated to explicitly describe the client configuration, partitioning strategy, aggregation procedure, communication protocol, and training settings, thereby substantially improving transparency, reproducibility, and comparability with existing federated learning studies.

**Comment:**
5-The Grad-CAM analysis was qualitative and lacked radiologist validation. Activation maps were described as highlighting "bone edges and joints" consistent with radiological markers, but no radiologist review of these maps was reported, no quantitative overlap with ground-truth anatomical regions was measured, and no examples of failure cases were shown. In a clinical context, selective presentation of successful Grad-CAM outputs without failure analysis was not sufficient to support interpretability claims.

**Response:**

We thank the reviewer for this important comment regarding clinical interpretability validation. The current Grad-CAM analysis was intended as an initial qualitative explainability assessment to visualize the discriminative regions influencing model predictions. We acknowledge that formal radiologist validation and quantitative localization metrics were not included in the original submission.

To address this limitation:

- Additional Grad-CAM visualizations across all classes have now been included,
- The manuscript explicitly states that the Grad-CAM analysis serves as a preliminary interpretability mechanism rather than definitive clinical validation.

**Comment:**
6-The abstract contained language issues and grammatical inconsistencies that indicated the manuscript needed language editing before resubmission. Phrases such as "a high value in the form of a macro F1 score" and "a technically superior solution" were inflated and imprecise. Several section headers and inline descriptions also shifted between describing osteoarthritis and osteoporosis as though they were interchangeable — they are distinct conditions, and the paper's focus should have been stated consistently throughout.

**Response:**

We sincerely thank the reviewer for highlighting the language and terminology inconsistencies present in the original manuscript. In response, the entire manuscript has undergone a comprehensive language revision to improve scientific readability, technical accuracy, grammatical consistency, and overall presentation quality. Several statements that could be perceived as subjective or overly promotional have been revised and replaced with objective scientific descriptions that more appropriately reflect the study findings. In particular, expressions such as “technically superior solution” and other inflated claims have been removed and rewritten using evidence-based language supported by the reported experimental results. Furthermore, a detailed terminology review was conducted throughout the manuscript to ensure consistency in the clinical context of the study. All incorrect references to osteoarthritis have been corrected to osteoporosis, which is the sole focus of the present research

**Reviewer #2:**

**Comment:**

1. Please specify the tools and methodologies used for image annotation. Additionally, clarify the criteria or validation process used to ensure annotation correctness and reliability.

**Response:**

We thank the reviewer for this important observation. The dataset utilized in this study consisted of clinically labeled knee X-ray images categorized into normal, osteopenia, and osteoporosis classes based on associated clinical diagnosis records. Therefore, manual pixel-level annotation was not required for the classification pipeline. For ROI localization experiments, YOLO-based automatic region extraction was applied to isolate the knee joint structures. The extracted regions were visually inspected to ensure anatomical correctness and consistency.

**Comment:**

2. The necessity and significance of performing Exploratory Data Analysis (EDA) in this study should be explained more clearly.

**Response:**

We appreciate the reviewer’s insightful comment. The Exploratory Data Analysis (EDA) was performed to:

1. Analyze class imbalance,
2. Examine feature distributions,
3. Identify missing values and inconsistencies,
4. Understand demographic and clinical relationships,
5. Validate the statistical characteristics of the dataset before model development.

The EDA additionally helped justify the need for ROI-based learning and class-balancing strategies by revealing substantial class imbalance and heterogeneous clinical distributions.

**Comment:**

3. The rational behind selecting the proposed OsteoNet-X architecture is not sufficiently justified. Please explain why this model was chosen over other existing single or hybrid deep learning models. Also, indicate whether comparative experiments were conducted using the same dataset.

**Response:**

We thank the reviewer for this comment. OsteoNet-X was selected after extensive comparative experimentation using multiple CNN backbones and optimization strategies on the same dataset. Preliminary experiments involving ResNet50, image-only CNNs, multimodal baselines, and federated configurations demonstrated limitations in feature generalization and minority-class sensitivity. The integration of YOLO-based ROI extraction further improved discriminative localization by focusing the model on clinically relevant knee joint structures.

DenseNet was ultimately selected due to:

- Improved feature propagation,
- Better gradient flow,
- Reduced parameter redundancy,
- Enhanced fine-grained texture representation.

**Comment:**
4. The Column Name Standardization in clinical data preprocessing methodology is not clearly described. In particular, the naming conventions and standardization of clinical data columns require clarification.

**Response:**

We appreciate the reviewer’s observation regarding the clinical data preprocessing procedure. To improve data consistency and ensure compatibility throughout the analytical pipeline, a systematic column name standardization process was performed prior to feature extraction and model development. Specifically, all column names were cleaned by removing leading and trailing whitespace characters, replacing internal spaces with underscore separators, eliminating special characters and punctuation symbols, and converting the naming format into a uniform structure. This standardization process ensured that feature names remained consistent across data loading, preprocessing, exploratory data analysis, and model training stages. Furthermore, it minimized potential parsing errors, prevented inconsistencies arising from heterogeneous naming conventions, and facilitated reliable feature mapping during subsequent image-clinical data integration and statistical analysis.

**Comment:**
5. Please define and explain the parameter RC used in Equation (4).

**Response:**

We thank the reviewer for identifying this omission. The parameter RC refers to the Region Confidence score generated during ROI localization. It represents the confidence probability associated with the detected knee joint region produced by the YOLO detector. The revised manuscript now explicitly defines RC and includes its mathematical interpretation within the methodology section.

**Comment:**
6. The process of obtaining the feature vector (fi) is unclear. Please provide a detailed explanation of the feature extraction procedure.

**Response:**

We appreciate the reviewer’s comment. The feature vector fi corresponds to the high-dimensional representation extracted from the final DenseNet global pooling layer after ROI-based feature encoding.

Specifically:

1. Input knee X-ray images are processed through convolutional and dense blocks,
2. Hierarchical feature maps are generated,
3. Global average pooling converts spatial feature maps into compact discriminative vectors,
4. The resulting vector fi is forwarded to the classification head.

A detailed mathematical explanation and feature extraction pipeline have now been added to the revised manuscript.

**Comment:**
7. The motivation for using a YOLO-based Region of Interest (ROI) extraction approach for the knee X-ray dataset is not clearly discussed prior to the Results section. Please justify its necessity and role in the proposed framework.

**Response:**

We thank the reviewer for this valuable observation. YOLO-based ROI extraction was incorporated to automatically localize anatomically relevant knee joint regions while suppressing irrelevant background structures. This significantly improves feature concentration and reduces noise during deep feature learning. The motivation for ROI extraction has now been explicitly introduced in the methodology section prior to the experimental analysis.

**Comment:**
8. The integration of YOLO-based ROI extraction, Deep Learning (DL), and Explainable AI (XAI) within the proposed framework is not sufficiently explained. Please clarify how these components are combined and the rationale behind this integration.

**Response:**

We sincerely thank the reviewer for this valuable observation. We agree that the interaction between the YOLO-based Region of Interest (ROI) localization module, the deep learning classification network, and the Explainable Artificial Intelligence (XAI) component was not sufficiently detailed in the original manuscript.

To improve clarity, a dedicated subsection has been added to the revised methodology in section 3.12 of the manuscript describing the complete integration workflow of the proposed OsteoNet-X framework. Specifically, the framework operates in three sequential stages. First, the YOLO-based ROI localization module automatically identifies and extracts the anatomically relevant knee joint region from the original radiograph, thereby reducing background redundancy and focusing the learning process on clinically meaningful structures. Second, the localized ROI is provided as input to the DenseNet-based OsteoNet-X classifier, where hierarchical feature representations are learned through densely connected convolutional layers and subsequently utilized for multi-class osteoporosis severity prediction. Finally, the Explainable AI module employs Gradient-weighted Class Activation Mapping (Grad-CAM) to generate visual attention maps that highlight the image regions contributing most significantly to the model’s decision.

The integration of these three components was intentionally designed to address three major challenges in osteoporosis image analysis: anatomical localization, discriminative feature learning, and model interpretability. The ROI localization stage improves feature quality by eliminating irrelevant image content, the DenseNet classifier enhances representation learning through dense feature propagation, and the Grad-CAM module provides visual explanations that support clinical trust and transparency. These additions have now been incorporated into the revised manuscript to provide a clear understanding of how localization, classification, and explainability are jointly utilized within the proposed OsteoNet-X framework.

**Comment:**
9. The manuscript lacks Grad-CAM visualization results. Please provide Grad-CAM or similar explain ability visualizations to demonstrate which regions of the knee X-ray images contribute most to the model’s predictions and to validate the clinical relevance of the proposed framework.

**Response:**

We thank the reviewer for this important suggestion. Grad-CAM visualization results have now been added for representative samples from all classes. The generated activation maps demonstrate that OsteoNet-X primarily focuses on clinically relevant knee joint regions and trabecular bone structures during classification.

**Comment:**
10. The manuscript currently provides limited visualization analysis. The authors are encouraged to implement and compare additional explainability or visualization techniques, apart from Grad-CAM, to further validate the robustness and interpretability of the proposed model.

**Response:**

We appreciate the reviewer’s important recommendation. In addition to Grad-CAM, the revised manuscript now discusses the potential integration of alternative explainability methods such as:

- Score-CAM,
- Eigen-CAM,
- SHAP-based visualization,
- Attention rollout mechanisms.

Due to computational limitations and manuscript scope constraints, Grad-CAM was selected as the primary explainability technique in this study. However, future work will incorporate multi-XAI comparative validation for enhanced interpretability.
