# Supplementary material for: OsteoNet-X: A deep residual learning framework for automated osteoporosis detection from knee X-ray images
Source: PLoS One. 2026 Oct 27;21(10):e0361033. doi: 10.1371/journal.pone.0361033 (PMC13712691; doi:10.1371/journal.pone.0361033)
Supplement: Supplementary file 2 [file pone.0361033.s003.docx]

**Response to the Reviewer Comments**

We sincerely thank the Editor and the Reviewers for their careful evaluation of our manuscript and for the constructive and insightful comments provided. We greatly appreciate the reviewers’ valuable suggestions, which have helped us improve the clarity, methodological transparency, technical rigor, and overall presentation of the manuscript. We have carefully considered all comments and have revised the manuscript accordingly.

All revisions have been incorporated into the revised manuscript, and the corresponding changes are highlighted/marked for ease of verification. Below, we provide a point-by-point response to each reviewer comment. We hope that the revisions adequately address the concerns raised and have substantially strengthened the quality and reproducibility of the work.

**Review #1 Comments to the Author**

**Comment 1: The limited sample size significantly restricts the generalizability of the findings to broader patient populations**.

**Response:**

We sincerely thank the reviewer for highlighting this important limitation. We fully agree that the relatively small sample size of 240 knee X-ray images limits the extent to which the reported performance can be generalized to broader and more heterogeneous patient populations. We have therefore revised the manuscript to present the reported results as evidence of performance on the evaluated dataset rather than as definitive evidence of clinical generalizability. The revised manuscript now explicitly states that the dataset size is substantially smaller than large-scale medical imaging benchmarks and that the possibility of dataset-specific learning and overfitting cannot be completely excluded. To mitigate this limitation during model development, the study employed transfer learning, class-weighted optimization, ROI-based localization, controlled fine-tuning, and training-only data augmentation. Importantly, augmentation was applied only to the training partitions and not to validation or test images, thereby avoiding artificial inflation of evaluation samples through leakage between partitions. The revised Discussion also emphasizes that independent external validation on larger, multi-institutional datasets is required before clinical deployment. We believe this clarification provides a more appropriately cautious interpretation of the 97.08% accuracy and 96.61% F1-score obtained on the present dataset.

**Comment:** **2-The manuscript exhibited substantial inconsistency between the initially described architecture and the final proposed framework. The title and abstract described OsteoNet-X as a "deep residual learning framework" using ResNet-based architecture, but the final model presented in the ablation study incorporated YOLO localization, DenseNet backbone, and Feature-Aware Learning optimization.**

**Response:**

We appreciate the reviewer for identifying this important inconsistency. We agree that the previous version could lead to ambiguity regarding the identity of the final proposed model. This issue has been comprehensively corrected. In the revised manuscript, OsteoNet-X is consistently defined as the final integrated framework consisting of YOLO-based Region of Interest localization, DenseNet-based hierarchical feature extraction and classification, task-specific optimization, and Grad-CAM-based explainability. The ResNet-based architecture is now explicitly described only as a baseline model used for comparative/preliminary experimentation and is no longer presented as the final OsteoNet-X architecture. The abstract, introduction, methodology, architecture figure, results, ablation study, discussion, and conclusion have been harmonized accordingly. We have also removed or rewritten statements that incorrectly characterize OsteoNet-X as a standalone residual network. The revised architecture figure now visually represents the actual final pipeline, thereby eliminating the previous discrepancy between the methodological description and the experimental results.

**Comment:** **3-**

**The binary classification experiments yielded substantially lower performance (57.92% accuracy) than the multi-class classification results (97.08% accuracy), an unexpected finding as binary classification tasks are generally easier than multi-class problems. The authors attributed this discrepancy to multimodal noise without providing sufficient empirical evidence or explanation for this counterintuitive phenomenon.**

**No binary classification results using the final optimized architecture (DenseNet + ROI) were reported, making it impossible to assess whether the performance gap reflected architectural differences or task-specific challenges.**

**Response:**

We thank the reviewer for this important observation. We agree that the original explanation was insufficient and that the difference between the preliminary binary result and the final multi-class result should not be interpreted as evidence that the binary task is intrinsically more difficult. The revised manuscript now clearly distinguishes the 57.92% binary result as an early image-only/baseline experiment rather than a result obtained using the final OsteoNet-X configuration. The multi-class result of 97.08% was obtained after the progressive architectural refinement involving ROI localization and the DenseNet-based backbone. Consequently, the two values are not directly comparable as controlled binary-versus-multi-class experiments because they differ in both task formulation and model configuration. We have removed the unsupported attribution to 'multimodal noise' and replaced it with a more defensible explanation centered on task formulation, class composition, architecture, and experimental configuration. We also clarify that the primary objective of the final framework is three-class severity assessment (Normal, Osteopenia, and Osteoporosis), rather than binary screening alone. Where a direct binary comparison using the final ROI + DenseNet configuration is reported, it should be presented as a controlled supplementary experiment using the same preprocessing, ROI extraction, backbone, and evaluation protocol.

**Comment: 4-Insufficiently Specified Federated Learning Configuration.**

**Response:**

We appreciate the reviewer for highlighting the need for a more detailed description of the federated learning configuration. We agree that the original manuscript did not provide sufficient implementation details to ensure reproducibility of the federated learning experiment. The revised manuscript now explicitly describes the client configuration, data partitioning strategy, local training procedure, global aggregation protocol, and communication settings.

The federated learning experiment was implemented as a simulation involving five virtual clients (K = 5), representing independent healthcare institutions. The dataset was distributed among the clients using a non-IID stratified partitioning strategy to simulate realistic variations in disease prevalence and patient distributions across participating institutions. Importantly, the patient images remained within their respective local client partitions during local training, while only model parameters were considered for global aggregation.

Each client locally trained the OsteoNet-X classification model for 3 local epochs using the AdamW optimizer with a batch size of 16. After completion of local training, the updated model parameters were transmitted to the central aggregation server. The server subsequently performed Federated Averaging (FedAvg) to obtain the updated global model. The contribution of each client was weighted according to the number of training samples available at that client.

The global training process was conducted for 20 communication rounds, with each round consisting of local model training followed by parameter aggregation. The revised manuscript now explicitly reports the following configuration: five clients, 20 communication rounds, 3 local epochs per round, batch size of 16, AdamW optimization, non-IID data partitioning, and FedAvg aggregation.

We further clarify that the federated learning experiment was designed as a supplementary privacy-preserving feasibility analysis, rather than as the primary training strategy used to obtain the main centralized classification results. Because the study involves a relatively small dataset, distributing the samples across multiple non-IID clients introduces additional statistical heterogeneity and reduces the amount of training data available to each local model. Consequently, differences between centralized and federated performance should be interpreted in the context of non-IID data distribution and limited sample size, rather than as an indication that the proposed architecture is unsuitable for federated deployment.

**Comment: 5-The Grad-CAM visualizations were presented qualitatively without formal radiologist validation, quantitative overlap metrics with ground-truth anatomical regions, or failure case analysis**

**Response:**

We thank the reviewer for this important limitation. We agree that Grad-CAM provides a model-attribution visualization rather than a clinical validation procedure, and that qualitative heatmaps alone cannot establish diagnostic validity. The revised manuscript therefore moderates the interpretation of the Grad-CAM results. The heatmaps are now presented as qualitative evidence of the regions contributing to model predictions rather than as proof of clinical correctness. We explicitly acknowledge that no formal radiologist rating study, anatomical ground-truth segmentation, quantitative localization metric such as Intersection-over-Union, or prospective clinical validation was performed in the present study. The Discussion and Limitations sections have been expanded to state that future work should involve independent radiologist assessment, predefined anatomical regions of interest, inter-rater agreement analysis, quantitative localization metrics, and systematic analysis of both successful and failure cases. We have also avoided language suggesting that Grad-CAM alone establishes clinical reliability.

**Comment: 6-The rationale for employing YOLO-based ROI extraction was not clearly established in the methodology section prior to the results presentation. The authors did not sufficiently justify why this specific approach was selected over alternative localization methods or demonstrate the necessity of ROI extraction for the proposed framework.**

**Response:**

We thank the reviewer for raising this important methodological concern. We agree that the motivation for incorporating Region of Interest (ROI) localization should have been established explicitly in the methodology before presenting the experimental results. The revised manuscript now provides a clear rationale for the use of YOLO-based knee ROI localization and distinguishes the motivation for ROI extraction from the subsequent classification architecture.

Knee X-ray images may contain anatomical structures, surrounding tissues, image borders, and background regions that are not directly relevant to osteoporosis severity classification. Since osteoporosis-related radiographic characteristics can be subtle and may be concentrated around anatomically relevant regions of the knee, providing the classifier with a localized knee region can reduce irrelevant visual information and allow the feature extractor to focus more specifically on the target anatomical structures.

YOLO was selected as the ROI localization mechanism because it provides single-stage object detection with direct bounding-box prediction, allowing the relevant knee region to be automatically localized without requiring manual cropping during inference. This makes the localization stage suitable for integration into an automated computer-aided diagnostic pipeline. Importantly, the revised manuscript does not claim that YOLO is universally superior to alternative localization techniques. Rather, YOLO was selected as a practical and computationally efficient localization approach for the proposed framework.

The usefulness of ROI localization was additionally investigated through the ablation experiments. In the reported ablation configuration, the image-only CNN achieved an accuracy of 28.33%, whereas the configuration incorporating YOLO-based ROI localization achieved 49.09% accuracy. This improvement provides dataset-specific empirical evidence that anatomical localization contributed positively to the classification pipeline. However, we have revised the interpretation to avoid attributing the entire improvement exclusively to ROI localization unless the compared configurations are otherwise identical.

The revised manuscript also explicitly separates the contributions of ROI localization, backbone architecture, task-specific optimization, federated learning, and Grad-CAM, ensuring that the ablation configurations are defined consistently throughout the methodology and results sections. Thus, the role of YOLO is presented specifically as an anatomical localization component rather than as a replacement for the DenseNet classifier or the federated learning configuration.

**Comment: 7-The integration of YOLO-based ROI localization, deep learning classification, and explainable AI components was not adequately explained. The manuscript lacked a clear description of how these three components were combined within the unified framework and the theoretical rationale supporting their synergistic integration.**

**Response:**

We thank the reviewer for this valuable observation. We agree that the previous manuscript did not sufficiently explain the functional relationship and information flow among the ROI localization, deep learning classification, and explainable artificial intelligence components. In the revised manuscript, we have therefore expanded the methodology to explicitly describe how these components operate as a unified sequential framework.

The proposed OsteoNet-X framework consists of three complementary functional stages: anatomical localization, discriminative classification, and post-hoc visual explanation. First, the preprocessed knee X-ray image is provided to the YOLO-based localization module. YOLO identifies the anatomically relevant knee joint region and generates a bounding box around the detected region. This bounding box is subsequently used to crop the input image, producing a localized ROI for subsequent analysis. The purpose of this stage is to reduce irrelevant background information and focus the classification network on the anatomical region of interest.

Second, the localized knee ROI is provided to the DenseNet-based classification module. DenseNet uses dense connectivity between successive convolutional layers, enabling feature reuse and hierarchical representation learning. The extracted feature maps are subsequently aggregated through Global Average Pooling and passed to the classification head. The final Softmax layer produces the predicted probability distribution across the three target categories: Normal, Osteopenia, and Osteoporosis. The task-specific optimization strategy is applied during training to improve the learning of discriminative class representations under the characteristics of the available dataset.

Third, Grad-CAM is applied to the trained DenseNet classifier as a post-hoc explainability mechanism. Grad-CAM uses the activations of the final convolutional feature representation together with the gradients associated with the predicted class to generate a class-discriminative heatmap. This heatmap is superimposed on the localized knee image to visually indicate the regions that contributed most strongly to the model's prediction.

Accordingly, the three components perform distinct but complementary functions within OsteoNet-X. YOLO performs anatomical localization, DenseNet performs hierarchical feature extraction and classification, and Grad-CAM provides visual attribution of the resulting prediction. The components are therefore not redundant: localization determines where the model should focus, DenseNet determines what visual representation should be learned for classification, and Grad-CAM provides an indication of which image regions contributed to the decision.

The revised architecture and methodology present in Figure 7 with page number 21, now explicitly represent this sequential information flow as:

**Input Knee X-ray → Image Preprocessing → YOLO-based ROI Localization → ROI Cropping → DenseNet Feature Extraction → Classification → Grad-CAM/XAI**

This integration is intended to provide a coherent image-analysis pipeline in which anatomical focusing precedes feature learning, while explainability is applied to the final classification process. We have also revised the architecture description and corresponding figure to ensure that the presented framework is consistent with the actual final OsteoNet-X implementation.

**Comment: 8-Limited Data Augmentation Description**

**Response:**

We thank the reviewer for identifying the insufficient description of the data augmentation protocol. We agree that the previous manuscript did not provide adequate details regarding the augmentation strategy or clearly specify how augmentation was handled during model development and evaluation.

In the revised manuscript, we have clarified that data augmentation is applied only to the training data. The validation and test partitions are maintained without augmentation so that model performance is evaluated on unseen, original images and to prevent information leakage between training and evaluation data.

The augmentation strategy consists of random horizontal flipping, small-angle rotation, controlled brightness and contrast variation, and the subsequent normalization required for pretrained convolutional networks. These transformations were selected to introduce moderate variations in image orientation and acquisition conditions while preserving the anatomical structures relevant to osteoporosis assessment. The transformations are applied stochastically during training rather than by creating a permanently expanded dataset.

Importantly, the revised manuscript distinguishes between the original dataset of 240 knee X-ray images and augmented training observations generated dynamically during model training. Augmented versions of an image are therefore not treated as additional independent patients or clinical observations. Similarly, validation and test samples are not augmented or duplicated for performance evaluation.

**Comment: 9-The manuscript contained language issues and grammatical inconsistencies throughout, with several instances of inflated claims lacking objective evidence. Additionally, terminology was used inconsistently, with the paper occasionally shifting between describing osteoarthritis and osteoporosis as if they were interchangeable conditions despite their distinct pathological characteristics.**

**Response:**

We sincerely thank the reviewer for highlighting this issue. The manuscript has undergone a comprehensive language and terminology revision. We have corrected grammatical errors, improved sentence structure, removed redundant expressions, and replaced inflated statements such as 'technically superior solution' or unsupported claims of clinical superiority with objective descriptions of the measured experimental findings. We have also standardized the disease terminology throughout the manuscript. The present study concerns osteoporosis, including the three diagnostic categories Normal, Osteopenia, and Osteoporosis; osteoarthritis is a distinct musculoskeletal condition and is not treated as a synonym for osteoporosis. All instances in the methodology, results, discussion, figure captions, and conclusion that incorrectly referred to osteoarthritis have been corrected to osteoporosis where appropriate. Claims of clinical usefulness have also been reframed as potential screening or decision-support applications that require external and prospective clinical validation before deployment.

**Reviewer 2 Comment:**

Reviewer #2: author made changes based on reviewer comments.
Author addressed all the queries raised by me.
can be accepted for publication.

**Response:**

We sincerely thank Reviewer #2 for the careful assessment of the revised manuscript and for confirming that the previous concerns have been adequately addressed. We are grateful for the constructive feedback, which helped us improve the methodological clarity, architectural consistency, reproducibility, and interpretability of the study.
