# Supplementary material for: OsteoNet-X: A deep residual learning framework for automated osteoporosis detection from knee X-ray images
Source: PLoS One. 2026 Oct 27;21(10):e0361033. doi: 10.1371/journal.pone.0361033 (PMC13712691; doi:10.1371/journal.pone.0361033)
Supplement: Peer review history

# Peer review: Decision Letter 0

**DOI:** 10.1371/journal.pone.0361033.r001

**Authors:** Gary S. Stein

14 May 2026

Dear Dr.  SANGULA,

Thank you for submitting your manuscript to PLOS ONE. After careful consideration, we feel that it has merit but does not fully meet PLOS ONE’s publication criteria as it currently stands. Therefore, we invite you to submit a revised version of the manuscript that addresses the points raised during the review process.

Please submit your revised manuscript by Jun 28 2026 11:59PM. If you will need more time than this to complete your revisions, please reply to this message or contact the journal office at plosone@plos.org. When you're ready to submit your revision, log on to https://www.editorialmanager.com/pone/ and select the 'Submissions Needing Revision' folder to locate your manuscript file.

- A letter that responds to each point raised by the academic editor and reviewer(s). You should upload this letter as a separate file labeled 'Response to Reviewers'.
- A marked-up copy of your manuscript that highlights changes made to the original version. You should upload this as a separate file labeled 'Revised Manuscript with Track Changes'.
- An unmarked version of your revised paper without tracked changes. You should upload this as a separate file labeled 'Manuscript'.

If you would like to make changes to your financial disclosure, please include your updated statement in your cover letter. Guidelines for resubmitting your figure files are available below the reviewer comments at the end of this letter.

If applicable, we recommend that you deposit your laboratory protocols in protocols.io to enhance the reproducibility of your results. Protocols.io assigns your protocol its own identifier (DOI) so that it can be cited independently in the future. For instructions see: https://journals.plos.org/plosone/s/submission-guidelines#loc-laboratory-protocols. Additionally, PLOS ONE offers an option for publishing peer-reviewed Lab Protocol articles, which describe protocols hosted on protocols.io. Read more information on sharing protocols at https://plos.org/protocols?utm_medium=editorial-email&utm_source=authorletters&utm_campaign=protocols.

As the corresponding author, your ORCID iD is verified in the submission system and will appear in the published article. PLOS supports the use of ORCID, and we encourage all coauthors to register for an ORCID iD and use it as well. Please encourage your coauthors to verify their ORCID iD within the submission system before final acceptance, as unverified ORCID iDs will not appear in the published article. *Only*  the individual author can complete the verification step; PLOS staff *cannot*  verify ORCID iDs on behalf of authors.

We look forward to receiving your revised manuscript.

Kind regards,

Gary S. Stein

Academic Editor

PLOS One

Journal Requirements:

When submitting your revision, we need you to address these additional requirements.

1.Please ensure that your manuscript meets PLOS ONE's style requirements, including those for file naming. The PLOS ONE style templates can be found at

https://journals.plos.org/plosone/s/file?id=wjVg/PLOSOne_formatting_sample_main_body.pdf and

https://journals.plos.org/plosone/s/file?id=ba62/PLOSOne_formatting_sample_title_authors_affiliations.pdf

2. Please note that PLOS One has specific guidelines on code sharing for submissions in which author-generated code underpins the findings in the manuscript. In these cases, we expect all author-generated code to be made available without restrictions upon publication of the work. Please review our guidelines at https://journals.plos.org/plosone/s/materials-and-software-sharing#loc-sharing-code and ensure that your code is shared in a way that follows best practice and facilitates reproducibility and reuse.

3. Please note that funding information should not appear in any section or other areas of your manuscript. We will only publish funding information present in the Funding Statement section of the online submission form. Please remove any funding-related text from the manuscript.

4. Thank you for uploading your study's underlying data set. Unfortunately, the repository you have noted in your Data Availability statement does not qualify as an acceptable data repository according to PLOS's standards.

At this time, please upload the minimal data set necessary to replicate your study's findings to a stable, public repository (such as figshare or Dryad) and provide us with the relevant URLs, DOIs, or accession numbers that may be used to access these data. For a list of recommended repositories and additional information on PLOS standards for data deposition, please see https://journals.plos.org/plosone/s/recommended-repositories.

5. Please amend the manuscript submission data (via Edit Submission) to include author “Dr.Siddique Ibrahim S.P.”

6. We note you have included a table to which you do not refer in the text of your manuscript. Please ensure that you refer to Table 3 and 4 in your text; if accepted, production will need this reference to link the reader to the Table.

7. If the reviewer comments include a recommendation to cite specific previously published works, please review and evaluate these publications to determine whether they are relevant and should be cited. There is no requirement to cite these works unless the editor has indicated otherwise.

[Note: HTML markup is below. Please do not edit.]

Reviewer's Responses to Questions

**Comments to the Author**

1. Is the manuscript technically sound, and do the data support the conclusions?

Reviewer #1: Partly

Reviewer #2: Yes

**********

2. Has the statistical analysis been performed appropriately and rigorously?

Reviewer #1: No

Reviewer #2: Yes

**********

3. Have the authors made all data underlying the findings in their manuscript fully available?

The PLOS Data policy

Reviewer #1: Yes

Reviewer #2: Yes

**********

4. Is the manuscript presented in an intelligible fashion and written in standard English?

Reviewer #1: Yes

Reviewer #2: Yes

**********

Reviewer #1: 1-The paper described two different models without clearly distinguishing them. The title and abstract described OsteoNet-X as a "deep residual learning framework" trained end-to-end, without ROI extraction or multimodal fusion. But the ablation study in Table 13 showed a completely different incremental pipeline: baseline CNN → YOLO ROI → Federated Learning → DenseNet backbone → Full OsteoNet-X. The full model in that table used YOLO localization and DenseNet, not a standalone ResNet. The introduction and methodology described one architecture; the results reported another. This inconsistency was never resolved in the paper, and it made it genuinely difficult to understand what OsteoNet-X actually was.

2-The dataset was critically small for the claims being made. The study used 240 knee X-ray images across three classes, with a test set inferred to be around 240 images for the without-augmentation condition (from the confusion matrix totals: 36 Normal, 154 Osteopenia, 50 Osteoporosis). Reporting 97.08% accuracy on a test set of this size — roughly 240 samples — did not constitute strong evidence of generalization. A model that memorized coarse class-level features could plausibly reach those numbers. The augmented dataset confusion matrix showed roughly 2,000 test images, suggesting augmentation substantially increased the effective test set size, but the augmentation protocol itself was not described: what transformations were applied, at what multiplicity, and whether augmented images were present in both training and test splits. If augmented images from the same original samples appeared in both, test performance would be inflated.

3-The binary classification results were substantially lower than the multi-class results, which was not explained. The image-only binary classification achieved an accuracy of only 57.92% (Table 3), while the final multi-class model achieved 97.08%. This was a counterintuitive finding — binary tasks are generally easier — and the paper attributed it to multimodal noise without providing evidence. No binary classification result using the same final architecture (DenseNet + ROI) was reported, making it impossible to assess whether the performance gap reflected the architecture or the task.

4-The federated learning simulation was not described with enough detail to be reproducible. The paper stated that data was "decentralized among multiple virtual clients" simulating independent healthcare facilities, but the number of clients, the data partitioning strategy, the aggregation algorithm (FedAvg or otherwise), the number of communication rounds, and the local training epochs were not specified. Without these details, the federated learning results in Table 10 could not be replicated or compared against established baselines.

5-The Grad-CAM analysis was qualitative and lacked radiologist validation. Activation maps were described as highlighting "bone edges and joints" consistent with radiological markers, but no radiologist review of these maps was reported, no quantitative overlap with ground-truth anatomical regions was measured, and no examples of failure cases were shown. In a clinical context, selective presentation of successful Grad-CAM outputs without failure analysis was not sufficient to support interpretability claims.

6-The abstract contained language issues and grammatical inconsistencies that indicated the manuscript needed language editing before resubmission. Phrases such as "a high value in the form of a macro F1 score" and "a technically superior solution" were inflated and imprecise. Several section headers and inline descriptions also shifted between describing osteoarthritis and osteoporosis as though they were interchangeable — they are distinct conditions, and the paper's focus should have been stated consistently throughout.

Reviewer #2: 1. Please specify the tools and methodologies used for image annotation. Additionally, clarify the criteria or validation process used to ensure annotation correctness and reliability.

2. The necessity and significance of performing Exploratory Data Analysis (EDA) in this study should be explained more clearly.

3. The rational behind selecting the proposed OsteoNet-X architecture is not sufficiently justified. Please explain why this model was chosen over other existing single or hybrid deep learning models. Also, indicate whether comparative experiments were conducted using the same dataset.

4. The Column Name Standardization in clinical data preprocessing methodology is not clearly described. In particular, the naming conventions and standardization of clinical data columns require clarification.

5. Please define and explain the parameter RC used in Equation (4).

6. The process of obtaining the feature vector (fi) is unclear. Please provide a detailed explanation of the feature extraction procedure.

7. The motivation for using a YOLO-based Region of Interest (ROI) extraction approach for the knee X-ray dataset is not clearly discussed prior to the Results section. Please justify its necessity and role in the proposed framework.

8. The integration of YOLO-based ROI extraction, Deep Learning (DL), and Explainable AI (XAI) within the proposed framework is not sufficiently explained. Please clarify how these components are combined and the rationale behind this integration.

9. The manuscript lacks Grad-CAM visualization results. Please provide Grad-CAM or similar explain ability visualizations to demonstrate which regions of the knee X-ray images contribute most to the model’s predictions and to validate the clinical relevance of the proposed framework.

10. The manuscript currently provides limited visualization analysis. The authors are encouraged to implement and compare additional explainability or visualization techniques, apart from Grad-CAM, to further validate the robustness and interpretability of the proposed model.

**********

what does this mean?). If published, this will include your full peer review and any attached files.

If you choose “no”, your identity will remain anonymous but your review may still be made public.

**Do you want your identity to be public for this peer review?**  For information about this choice, including consent withdrawal, please see our Privacy Policy

Reviewer #1: No

Reviewer #2: **Yes:**  Dr. B.Surendiran

**********

[NOTE: If reviewer comments were submitted as an attachment file, they will be attached to this email and accessible via the submission site. Please log into your account, locate the manuscript record, and check for the action link "View Attachments". If this link does not appear, there are no attachment files.]

To ensure your figures meet our technical requirements, please review our figure guidelines: https://journals.plos.org/plosone/s/figures

You may also use PLOS’s free figure tool, NAAS, to help you prepare publication quality figures: https://journals.plos.org/plosone/s/figures#loc-tools-for-figure-preparation.

NAAS will assess whether your figures meet our technical requirements by comparing each figure against our figure specifications.

# Author comment: Author response to Decision Letter 1

**DOI:** 10.1371/journal.pone.0361033.r002

9 Jul 2026

We thank the Academic Editor and reviewers for their valuable comments and suggestions. We have carefully revised the manuscript and addressed all concerns raised during the review process. Specifically, we have updated the Data Availability Statement and deposited the dataset in a public Figshare repository (DOI: 10.6084/m9.figshare.32593518). In accordance with PLOS ONE guidelines, the source code has been made publicly available through GitHub (https://github.com/sangvisudhir-svg/OsteoNet-X-Knee-Xray-Osteoporosis-Classification). We have also revised the manuscript to include explicit references to Tables 3 and 4, improved the discussion of the results, and corrected formatting and presentation issues throughout the manuscript. A detailed point-by-point response to all editor and reviewer comments has been provided in the uploaded "Response to Reviewers" document.

Attachment Submitted filename: Response to Reviewers.docx

# Peer review: Decision Letter 1

**DOI:** 10.1371/journal.pone.0361033.r003

**Authors:** Gary S. Stein

5 Aug 2026

Dear Dr.  S P,

Thank you for submitting your manuscript to PLOS ONE. After careful consideration, we feel that it has merit but does not fully meet PLOS ONE’s publication criteria as it currently stands. Therefore, we invite you to submit a revised version of the manuscript that addresses the points raised during the review process.

Please submit your revised manuscript by Sep 19 2026 11:59PM. If you will need more time than this to complete your revisions, please reply to this message or contact the journal office at plosone@plos.org. When you're ready to submit your revision, log on to https://www.editorialmanager.com/pone/ and select the 'Submissions Needing Revision' folder to locate your manuscript file.

- A letter that responds to each point raised by the academic editor and reviewer(s). You should upload this letter as a separate file labeled 'Response to Reviewers'.
- A marked-up copy of your manuscript that highlights changes made to the original version. You should upload this as a separate file labeled 'Revised Manuscript with Track Changes'.
- An unmarked version of your revised paper without tracked changes. You should upload this as a separate file labeled 'Manuscript'.

If you would like to make changes to your financial disclosure, please include your updated statement in your cover letter. Guidelines for resubmitting your figure files are available below the reviewer comments at the end of this letter.

If applicable, we recommend that you deposit your laboratory protocols in protocols.io to enhance the reproducibility of your results. Protocols.io assigns your protocol its own identifier (DOI) so that it can be cited independently in the future. For instructions see: https://journals.plos.org/plosone/s/submission-guidelines#loc-laboratory-protocols. Additionally, PLOS ONE offers an option for publishing peer-reviewed Lab Protocol articles, which describe protocols hosted on protocols.io. Read more information on sharing protocols at https://plos.org/protocols?utm_medium=editorial-email&utm_source=authorletters&utm_campaign=protocols.

As the corresponding author, your ORCID iD is verified in the submission system and will appear in the published article. PLOS supports the use of ORCID, and we encourage all coauthors to register for an ORCID iD and use it as well. Please encourage your coauthors to verify their ORCID iD within the submission system before final acceptance, as unverified ORCID iDs will not appear in the published article. *Only*  the individual author can complete the verification step; PLOS staff *cannot*  verify ORCID iDs on behalf of authors.

We look forward to receiving your revised manuscript.

Kind regards,

Gary S. Stein

Academic Editor

PLOS One

Journal Requirements:

If the reviewer comments include a recommendation to cite specific previously published works, please review and evaluate these publications to determine whether they are relevant and should be cited. There is no requirement to cite these works unless the editor has indicated otherwise.

[Note: HTML markup is below. Please do not edit.]

Reviewers' comments:

Reviewer's Responses to Questions

**Comments to the Author**

Reviewer #1: All comments have been addressed

Reviewer #2: All comments have been addressed

**********

2. Is the manuscript technically sound, and do the data support the conclusions?

Reviewer #1: Partly

Reviewer #2: Yes

**********

3. Has the statistical analysis been performed appropriately and rigorously?

Reviewer #1: No

Reviewer #2: N/A

**********

4. Have the authors made all data underlying the findings in their manuscript fully available?

The PLOS Data policy

Reviewer #1: Yes

Reviewer #2: Yes

**********

5. Is the manuscript presented in an intelligible fashion and written in standard English?

Reviewer #1: No

Reviewer #2: Yes

**********

Reviewer #1: 1- the limited sample size significantly restricts the generalizability of the findings to broader patient populations.

2-The manuscript exhibited substantial inconsistency between the initially described architecture and the final proposed framework. The title and abstract described OsteoNet-X as a "deep residual learning framework" using ResNet-based architecture, but the final model presented in the ablation study incorporated YOLO localization, DenseNet backbone, and Feature-Aware Learning optimization.

3-The binary classification experiments yielded substantially lower performance (57.92% accuracy) than the multi-class classification results (97.08% accuracy), an unexpected finding as binary classification tasks are generally easier than multi-class problems. The authors attributed this discrepancy to multimodal noise without providing sufficient empirical evidence or explanation for this counterintuitive phenomenon. No binary classification results using the final optimized architecture (DenseNet + ROI) were reported, making it impossible to assess whether the performance gap reflected architectural differences or task-specific challenges

4-Insufficiently Specified Federated Learning Configuration.

5-The Grad-CAM visualizations were presented qualitatively without formal radiologist validation, quantitative overlap metrics with ground-truth anatomical regions, or failure case analysis

6-The rationale for employing YOLO-based ROI extraction was not clearly established in the methodology section prior to the results presentation. The authors did not sufficiently justify why this specific approach was selected over alternative localization methods or demonstrate the necessity of ROI extraction for the proposed framework.

7-The integration of YOLO-based ROI localization, deep learning classification, and explainable AI components was not adequately explained. The manuscript lacked a clear description of how these three components were combined within the unified framework and the theoretical rationale supporting their synergistic integration.

8-Limited Data Augmentation Description

9-The manuscript contained language issues and grammatical inconsistencies throughout, with several instances of inflated claims lacking objective evidence. Additionally, terminology was used inconsistently, with the paper occasionally shifting between describing osteoarthritis and osteoporosis as if they were interchangeable conditions despite their distinct pathological characteristics.

Reviewer #2: author made changes based on reviewer comments.

Author addressed all the queries raised by me.

can be accepted for publication.

**********

what does this mean?). If published, this will include your full peer review and any attached files.

If you choose “no”, your identity will remain anonymous but your review may still be made public.

**Do you want your identity to be public for this peer review?**  For information about this choice, including consent withdrawal, please see our Privacy Policy

Reviewer #1: No

Reviewer #2: No

**********

[NOTE: If reviewer comments were submitted as an attachment file, they will be attached to this email and accessible via the submission site. Please log into your account, locate the manuscript record, and check for the action link "View Attachments". If this link does not appear, there are no attachment files.]

To ensure your figures meet our technical requirements, please review our figure guidelines: https://journals.plos.org/plosone/s/figures

You may also use PLOS’s free figure tool, NAAS, to help you prepare publication quality figures: https://journals.plos.org/plosone/s/figures#loc-tools-for-figure-preparation.

NAAS will assess whether your figures meet our technical requirements by comparing each figure against our figure specifications.

# Author comment: Author response to Decision Letter 2

**DOI:** 10.1371/journal.pone.0361033.r004

18 Aug 2026

Response to the Reviewer Comments

We sincerely thank the Editor and the Reviewers for their careful evaluation of our manuscript and for the constructive and insightful comments provided. We greatly appreciate the reviewers’ valuable suggestions, which have helped us improve the clarity, methodological transparency, technical rigor, and overall presentation of the manuscript. We have carefully considered all comments and have revised the manuscript accordingly.

All revisions have been incorporated into the revised manuscript, and the corresponding changes are highlighted/marked for ease of verification. Below, we provide a point-by-point response to each reviewer comment. We hope that the revisions adequately address the concerns raised and have substantially strengthened the quality and reproducibility of the work.

Review #1 Comments to the Author

Comment 1: The limited sample size significantly restricts the generalizability of the findings to broader patient populations.

Response:

We sincerely thank the reviewer for highlighting this important limitation. We fully agree that the relatively small sample size of 240 knee X-ray images limits the extent to which the reported performance can be generalized to broader and more heterogeneous patient populations. We have therefore revised the manuscript to present the reported results as evidence of performance on the evaluated dataset rather than as definitive evidence of clinical generalizability. The revised manuscript now explicitly states that the dataset size is substantially smaller than large-scale medical imaging benchmarks and that the possibility of dataset-specific learning and overfitting cannot be completely excluded. To mitigate this limitation during model development, the study employed transfer learning, class-weighted optimization, ROI-based localization, controlled fine-tuning, and training-only data augmentation. Importantly, augmentation was applied only to the training partitions and not to validation or test images, thereby avoiding artificial inflation of evaluation samples through leakage between partitions. The revised Discussion also emphasizes that independent external validation on larger, multi-institutional datasets is required before clinical deployment. We believe this clarification provides a more appropriately cautious interpretation of the 97.08% accuracy and 96.61% F1-score obtained on the present dataset.

Comment: 2-The manuscript exhibited substantial inconsistency between the initially described architecture and the final proposed framework. The title and abstract described OsteoNet-X as a "deep residual learning framework" using ResNet-based architecture, but the final model presented in the ablation study incorporated YOLO localization, DenseNet backbone, and Feature-Aware Learning optimization.

Response:

We appreciate the reviewer for identifying this important inconsistency. We agree that the previous version could lead to ambiguity regarding the identity of the final proposed model. This issue has been comprehensively corrected. In the revised manuscript, OsteoNet-X is consistently defined as the final integrated framework consisting of YOLO-based Region of Interest localization, DenseNet-based hierarchical feature extraction and classification, task-specific optimization, and Grad-CAM-based explainability. The ResNet-based architecture is now explicitly described only as a baseline model used for comparative/preliminary experimentation and is no longer presented as the final OsteoNet-X architecture. The abstract, introduction, methodology, architecture figure, results, ablation study, discussion, and conclusion have been harmonized accordingly. We have also removed or rewritten statements that incorrectly characterize OsteoNet-X as a standalone residual network. The revised architecture figure now visually represents the actual final pipeline, thereby eliminating the previous discrepancy between the methodological description and the experimental results.

Comment: 3-

The binary classification experiments yielded substantially lower performance (57.92% accuracy) than the multi-class classification results (97.08% accuracy), an unexpected finding as binary classification tasks are generally easier than multi-class problems. The authors attributed this discrepancy to multimodal noise without providing sufficient empirical evidence or explanation for this counterintuitive phenomenon.

No binary classification results using the final optimized architecture (DenseNet + ROI) were reported, making it impossible to assess whether the performance gap reflected architectural differences or task-specific challenges.

Response:

We thank the reviewer for this important observation. We agree that the original explanation was insufficient and that the difference between the preliminary binary result and the final multi-class result should not be interpreted as evidence that the binary task is intrinsically more difficult. The revised manuscript now clearly distinguishes the 57.92% binary result as an early image-only/baseline experiment rather than a result obtained using the final OsteoNet-X configuration. The multi-class result of 97.08% was obtained after the progressive architectural refinement involving ROI localization and the DenseNet-based backbone. Consequently, the two values are not directly comparable as controlled binary-versus-multi-class experiments because they differ in both task formulation and model configuration. We have removed the unsupported attribution to 'multimodal noise' and replaced it with a more defensible explanation centered on task formulation, class composition, architecture, and experimental configuration. We also clarify that the primary objective of the final framework is three-class severity assessment (Normal, Osteopenia, and Osteoporosis), rather than binary screening alone. Where a direct binary comparison using the final ROI + DenseNet configuration is reported, it should be presented as a controlled supplementary experiment using the same preprocessing, ROI extraction, backbone, and evaluation protocol.

Comment: 4-Insufficiently Specified Federated Learning Configuration.

Response:

We appreciate the reviewer for highlighting the need for a more detailed description of the federated learning configuration. We agree that the original manuscript did not provide sufficient implementation details to ensure reproducibility of the federated learning experiment. The revised manuscript now explicitly describes the client configuration, data partitioning strategy, local training procedure, global aggregation protocol, and communication settings.

The federated learning experiment was implemented as a simulation involving five virtual clients (K = 5), representing independent healthcare institutions. The dataset was distributed among the clients using a non-IID stratified partitioning strategy to simulate realistic variations in disease prevalence and patient distributions across participating institutions. Importantly, the patient images remained within their respective local client partitions during local training, while only model parameters were considered for global aggregation.

Each client locally trained the OsteoNet-X classification model for 3 local epochs using the AdamW optimizer with a batch size of 16. After completion of local training, the updated model parameters were transmitted to the central aggregation server. The server subsequently performed Federated Averaging (FedAvg) to obtain the updated global model. The contribution of each client was weighted according to the number of training samples available at that client.

The global training process was conducted for 20 communication rounds, with each round consisting of local model training followed by parameter aggregation. The revised manuscript now explicitly reports the following configuration: five clients, 20 communication rounds, 3 local epochs per round, batch size of 16, AdamW optimization, non-IID data partitioning, and FedAvg aggregation.

We further clarify that the federated learning experiment was designed as a supplementary privacy-preserving feasibility analysis, rather than as the primary training strategy used to obtain the main centralized classification results. Because the study involves a relatively small dataset, distributing the samples across multiple non-IID clients introduces additional statistical heterogeneity and reduces the amount of training data available to each local model. Consequently, differences between centralized and federated performance should be interpreted in the context of non-IID data distribution and limited sample size, rather than as an indication that the proposed architecture is unsuitable for federated deployment.

Comment: 5-The Grad-CAM visualizations were presented qualitatively without formal radiologist validation, quantitative overlap metrics with ground-truth anatomical regions, or failure case analysis

Response:

We thank the reviewer for this important limitation. We agree that Grad-CAM provides a model-attribution visualization rather than a clinical validation procedure, and that qualitative heatmaps alone cannot establish diagnostic validity. The revised manuscript therefore moderates the interpretation of the Grad-CAM results. The heatmaps are now presented as qualitative evidence of the regions contributing to model predictions rather than as proof of clinical correctness. We explicitly acknowledge that no formal radiologist rating study, anatomical ground-truth segmentation, quantitative localization metric such as Intersection-over-Union, or prospective clinical validation was performed in the present study. The Discussion and Limitations sections have been expanded to state that future work should involve independent radiologist assessment, predefined anatomical regions of interest, inter-rater agreement analysis, quantitative localization metrics, and systematic analysis of both successful and failure cases. We have also avoided language suggesting that Grad-CAM alone establishes clinical reliability.

Comment: 6-The rationale for employing YOLO-based ROI extraction was not clearly established in the methodology section prior to the results presentation. The authors did not sufficiently justify why this specific approach was selected over alternative localization methods or demonstrate the necessity of ROI extraction for the proposed framework.

Response:

We thank the reviewer for raising this important methodological concern. We agree that the motivation for incorporating Region of Interest (ROI) localization should have been established explicitly in the methodology before presenting the experimental results. The revised manuscript now provides a clear rationale for the use of YOLO-based knee ROI localization and distinguishes the motivation for ROI extraction from the subsequent classification architecture.

Knee X-ray images may contain anatomical structures, surrounding tissues, image borders, and background regions that are not directly relevant to osteoporosis severity classification. Since osteoporosis-related radiographic characteristics can be subtle and may be concentrated around anatomically relevant regions of the knee, providing the classifier with a localized knee region can reduce irrelevant visual information and allow the feature extractor to focus more specifically on the target anatomical structures.

YOLO was selected as the ROI localization mechanism because it provides single-stage object detection with direct bounding-box prediction, allowing the relevant knee region to be automatically localized without requiring manual cropping during inference. This makes the localization stage suitable for integration into an automated computer-aided diagnostic pipeline. Importantly, the revised manuscript does not claim that YOLO is universally superior to alternative localization techniques. Rather, YOLO was selected as a practical and computationally efficient localization approach for the proposed framework.

The usefulness of ROI localization was additionally investigated through the ablation experiments. In the reported ablation configuration, the image-only CNN achieved an accuracy of 28.33%, whereas the configuration incorporating YOLO-based ROI localization achieved 49.09% accuracy. This improvement provides dataset-specific empirical evidence that anatomical localization contributed positively to the classification pipeline. However, we have revised the interpretation to avoid attributing the entire improvement exclusively to ROI localization unless the compared configurations are otherwise identical.

The revised manuscript also explicitly separates the contributions of ROI localization, backbone architecture, task-specific optimization, federated learning, and Grad-CAM, ensuring that the ablation configurations are defined consistently throughout the methodology and results sections. Thus, the role of YOLO is presented specifically as an anatomical localization component rather than as a replacement for the DenseNet classifier or the federated learning configuration.

Comment: 7-The integration of YOLO-based ROI localization, deep learning classification, and explainable AI components was not adequately explained. The manuscript lacked a clear description of how these three components were combined within the unified framework and the theoretical rationale supporting their synergistic integration.

Response:

We thank the reviewer for this valuable observation. We agree that the previous manuscript did not sufficiently explain the functional relationship and information flow among the ROI localization, deep learning classification, and explainable artificial intelligence components. In the revised manuscript, we have therefore expanded the methodology to explicitly describe how these components operate as a unified sequential framework.

The proposed OsteoNet-X framework consists of three complementary functional stages: anatomical localization, discriminative classification, and post-hoc visual explanation. First, the preprocessed knee X-ray image is provided to the YOLO-based localization module. YOLO identifies the anatomically relevant knee joint region and generates a bounding box around the detected region. This bounding box is subsequently used to crop the input image, producing a localized ROI for subsequent analysis. The purpose of this stage is to reduce irrelevant background information and focus the classification network on the anatomical region of interest.

Second, the localized knee ROI is provided to the DenseNet-based classification module. DenseNet uses dense connectivity between successive convolutional layers, enabling feature reuse and hierarchical representation learning. The extracted feature maps are subsequently aggregated through Global Average Pooling and passed to the classification head. The final Softmax layer produces the predicted probability distribution across the three target categories: Normal, Osteopenia, and Osteoporosis. The task-specific optimization strategy is applied during training to improve the learning of discriminative class representations under the characteristics of the available dataset.

Third, Grad-CAM is applied to the trained DenseNet classifier as a post-hoc explainability mechanism. Grad-CAM uses the activations of the final convolutional feature representation together with the gradients associated with the predicted class to generate a class-discriminative heatmap. This heatmap is superimposed on the localized knee image to visually indicate the regions that contributed most strongly to the model's prediction.

Accordingly, the three components perform distinct but complementary functions within OsteoNet-X. YOLO performs anatomical localization, DenseNet performs hierarchical feature extraction and classification, and Grad-CAM provides visual attribution of the resulting prediction. The components are therefore not redundant: localization determines where the model should focus, DenseNet determines what visual representation should be learned for classification, and Grad-CAM provides an indication of which image regions contributed to the decision.

The revised architecture and methodology present in Figure 7 with page number 21, now explicitly represent this sequential information flow as:

Input Knee X-ray → Image Preprocessing → YOLO-based ROI Localization → ROI Cropping → DenseNet Feature Extraction → Classification → Grad-CAM/XAI

This integration is intended to provide a coherent image-analysis pipeline in which anatomical focusing precedes feature learning, while explainability is applied to the final classification process. We have also revised the architecture description and corresponding figure to ensure that the presented framework is consistent with the actual final OsteoNet-X implementation.

Comment: 8-Limited Data Augmentation Description

Response:

We thank the reviewer for identifying the insufficient description of the data augmentation protocol. We agree that the previous manuscript did not provide adequate details regarding the augmentation strategy or clearly specify how augmentation was handled during model development and evaluation.

In the revised manuscript, we have clarified that data augmentation is applied only to the training data. The validation and test partitions are maintained without augmentation so that model performance is evaluated on unseen, original images and to prevent information leakage between training and evaluation data.

The augmentation strategy consists of random horizontal flipping, small-angle rotation, controlled brightness and contrast variation, and the subsequent normalization required for pretrained convolutional networks. These transformations were selected to introduce moderate variations in image orientation and acquisition conditions while preserving the anatomical structures relevant to osteoporosis assessment. The transformations are applied stochastically during training rather than by creating a permanently expanded dataset.

Importantly, the revised manuscript distinguishes between the original dataset of 240 knee X-ray images and augmented training observations generated dynamically during model training. Augmented versions of an image are therefore not treated as additional independent patients or clinical observations. Similarly, validation and test samples are not augmented or duplicated for performance evaluation.

Comment: 9-The manuscript contained language issues and grammatical inconsistencies throughout, with several instances of inflated claims lacking objective evidence. Additionally, terminology was used inconsistently, with the paper occasionally shifting between describing osteoarthritis and osteoporosis as if they were interchangeable conditions despite their distinct pathological characteristics.

Response:

We sincerely thank the reviewer for highlighting this issue. The manuscript has undergone a comprehensive language and terminology revision. We have corrected grammatical errors, improved sentence structure, removed redundant expressions, and replaced inflated statements such as 'technically superior solution' or unsupported claims of clinical superiority with objective descriptions of the measured experimental findings. We have also standardized the disease terminology throughout the manuscript. The present study concerns osteoporosis, including the three diagnostic categories Normal, Osteopenia, and Osteoporosis; osteoarthritis is a distinct musculoskeletal condition and is not treated as a synonym for osteoporosis. All instances in the methodology, results, discussion, figure captions, and conclusion that incorrectly referred to osteoarthritis have been corrected to osteoporosis where appropriate. Claims of clinical usefulness have also been reframed as potential screening or decision-support applications that require external and prospective clinical validation before deployment.

Reviewer 2 Comment:

Reviewer #2: author made changes based on reviewer comments.

Author addressed all the queries raised by me.

can be accepted for publication.

Response:

We sincerely thank Reviewer #2 for the careful assessment of the revised manuscript and for confirming that the previous concerns have been adequately addressed. We are grateful for the constructive feedback, which helped us improve the methodological clarity, architectural consistency, reproducibility, and interpretability

Attachment Submitted filename: Response_to_Reviewers_auresp_2.docx

# Peer review: Decision Letter 2

**DOI:** 10.1371/journal.pone.0361033.r005

**Authors:** Gary S. Stein

6 Oct 2026

OsteoNet-X: A Deep Residual Learning Framework for Automated Osteoporosis Detection from Knee X-ray Images

PONE-D-26-11503R2

Dear Dr. Ibrahim,

We’re pleased to inform you that your manuscript has been judged scientifically suitable for publication and will be formally accepted for publication once it meets all outstanding technical requirements.

Within one week, you’ll receive an e-mail detailing the required amendments. When these have been addressed, you’ll receive a formal acceptance letter and your manuscript will be scheduled for publication.

An invoice will be generated when your article is formally accepted. Please note, if your institution has a publishing partnership with PLOS and your article meets the relevant criteria, all or part of your publication costs will be covered. Please make sure your user information is up-to-date by logging into Editorial Manager at Editorial Manager® and clicking the ‘Update My Information' link at the top of the page. For questions related to billing, please contact billing support.

If your institution or institutions have a press office, please notify them about your upcoming paper to help maximize its impact. If they’ll be preparing press materials, please inform our press team as soon as possible -- no later than 48 hours after receiving the formal acceptance. Your manuscript will remain under strict press embargo until 2 pm Eastern Time on the date of publication. For more information, please contact onepress@plos.org.

Kind regards,

Gary S. Stein

Academic Editor

PLOS One

**Comments to the Author**

Reviewer #1: All comments have been addressed

Reviewer #2: All comments have been addressed

**********

2. Is the manuscript technically sound, and do the data support the conclusions?

Reviewer #1: Yes

Reviewer #2: Yes

**********

3. Has the statistical analysis been performed appropriately and rigorously?

Reviewer #1: N/A

Reviewer #2: Yes

**********

4. Have the authors made all data underlying the findings in their manuscript fully available?

The PLOS Data policy

Reviewer #1: Yes

Reviewer #2: Yes

**********

5. Is the manuscript presented in an intelligible fashion and written in standard English?

Reviewer #1: Yes

Reviewer #2: Yes

**********

Reviewer #1: This revised manuscript presents a deep learning framework for multi-class osteoporosis severity classification from knee X-ray images. The authors have made substantial revisions in response to prior reviewer comments, including clarifying the architectural ambiguity (ResNet vs. DenseNet), repositioning the federated learning experiment as supplementary, and moderating claims regarding clinical validity. The manuscript is generally well-written and the revisions demonstrate careful attention to reviewer feedback.

Reviewer #2: author made changes based on review comments . can be accepted for publication.

author made changes based on review comments . can be accepted for publication.

**********

what does this mean?). If published, this will include your full peer review and any attached files.

If you choose “no”, your identity will remain anonymous but your review may still be made public.

**Do you want your identity to be public for this peer review?**  For information about this choice, including consent withdrawal, please see our Privacy Policy

Reviewer #1: No

Reviewer #2: No

**********

# Editor report: Acceptance letter

**DOI:** 10.1371/journal.pone.0361033.r006

**Authors:** Gary S. Stein

PONE-D-26-11503R2

PLOS One

Dear Dr. S.P.,

I'm pleased to inform you that your manuscript has been deemed suitable for publication in PLOS One. Congratulations! Your manuscript is now being handed over to our production team.

At this stage, our production department will prepare your paper for publication. This includes ensuring the following:

* All references, tables, and figures are properly cited

* All relevant supporting information is included in the manuscript submission,

* There are no issues that prevent the paper from being properly typeset

You will receive further instructions from the production team, including instructions on how to review your proof when it is ready. Please keep in mind that we are working through a large volume of accepted articles, so please give us a few days to review your paper and let you know the next and final steps.

Lastly, if your institution or institutions have a press office, please let them know about your upcoming paper now to help maximize its impact. If they'll be preparing press materials, please inform our press team within the next 48 hours. Your manuscript will remain under strict press embargo until 2 pm Eastern Time on the date of publication. For more information, please contact onepress@plos.org.

You will receive an invoice from PLOS for your publication fee after your manuscript has reached the completed accept phase. If you receive an email requesting payment before acceptance or for any other service, this may be a phishing scheme. Learn how to identify phishing emails and protect your accounts at https://explore.plos.org/phishing.

If we can help with anything else, please email us at customercare@plos.org.

Thank you for submitting your work to PLOS One and supporting open access.

Kind regards,

PLOS One Editorial Office Staff

on behalf of

Dr. Gary S. Stein

Academic Editor

PLOS One
